# Wnt and Neuregulin1/ErbB signalling extends 3D culture of hormone responsive mammary organoids

**DOI:** 10.1038/ncomms13207

**Published:** 2016-10-26

**Authors:** Thierry Jardé, Bethan Lloyd-Lewis, Mairian Thomas, Howard Kendrick, Lorenzo Melchor, Lauriane Bougaret, Peter D. Watson, Kenneth Ewan, Matthew J. Smalley, Trevor C. Dale

**Affiliations:** 1 Cardiff School of Biosciences, Cardiff University, Cardiff CF10 3AX, UK; 2 Cancer Program, Development and Stem Cells Program, Monash Biomedicine Discovery Institute, Clayton, Victoria 3800, Australia; 3 Department of Anatomy and Developmental Biology, Monash University, Clayton, Victoria 3800, Australia; 4 Centre for Cancer Research, Hudson Institute of Medical Research, Clayton, Victoria 3168, Australia; 5 Department of Pathology, University of Cambridge, Cambridge CB2 1QP, UK; 6 European Cancer Stem Cell Research Institute, Cardiff School of Biosciences, Cardiff University, Cardiff CF24 4HQ, UK; 7 Division of Breast Cancer Research, Breast Cancer Now, Institute of Cancer Research, London SW3 6JB, UK

## Abstract

The development of *in vitro* culture systems quantitatively and qualitatively recapitulating normal breast biology is key to the understanding of mammary gland biology. Current three-dimensional mammary culture systems have not demonstrated concurrent proliferation and functional differentiation *ex vivo* in any system for longer than 2 weeks. Here, we identify conditions including Neuregulin1 and R-spondin 1, allowing maintenance and expansion of mammary organoids for 2.5 months in culture. The organoids comprise distinct basal and luminal compartments complete with functional steroid receptors and stem/progenitor cells able to reconstitute a complete mammary gland *in vivo.* Alternative conditions are also described that promote enrichment of basal cells organized into multiple layers surrounding a keratinous core, reminiscent of structures observed in MMTV-Wnt1 tumours. These conditions comprise a unique tool that should further understanding of normal mammary gland development, the molecular mechanism of hormone action and signalling events whose deregulation leads to breast tumourigenesis.

## Abstract (web summary)

Three-dimensional culture systems and organoids for mammary glands are important to understand mammary gland development. Here, the authors identify conditions (including Neuregulin 1 and R-spondin 1) that allow the culture of organoids that are responsive to hormonal stimulation for up to 2.5 months.

A detailed knowledge of normal mammary gland development and the mechanisms driving its molecular, cellular and hormonal regulation, is fundamental to an understanding of the initiation and progression of breast cancer. The mammary gland consists of an elaborate, tree-like network of branched ducts and lobular alveolar structures, embedded within a stromal fat pad. Bilayered ductal and alveolar structures possess an inner layer of luminal epithelial cells surrounding a central lumen, and an outer layer of basal cells, enveloped by a laminin-rich basement membrane separating the parenchymal and stromal compartments. The luminal cell layer is composed of two functionally distinct lineages defined by the expression or absence of steroid hormone receptors. The basal cell population consists of myoepithelial cells with contractile properties and presumptive multipotent mammary stem cells, although distinct unipotent stem cells committed to either luminal or basal lineages have also been reported^1^,^2^.

The development of three-dimensional (3D) mammary gland *in vitro* culture systems has contributed greatly to the understanding of mammary gland biology, offering insights into cell–cell interactions, paracrine signalling, cell proliferation, differentiation and hormonal regulation^3^,^4^,^5^. Furthermore, research into the stem cell niche and breast carcinogenesis has been facilitated by the ability to culture complex multicellular mammary structures in 3D. Thus far, however, studies have yet to establish culture conditions concurrently enabling sustained proliferation, stem cell maintenance and functional differentiation in tissues *ex vivo* for extended periods. For example, a recent strategy combining Wnt-3a-mediated Wnt signalling activation and epidermal growth factor (EGF) treatment allows long-term expansion of murine mammary stem cells able to form small, disorganized round colonies in 3D culture^6^. In contrast, a cocktail of biological factors including insulin, EGF or fibroblast growth factor allow the short-term maintenance of polarized epithelial cells surrounding a lumen that contains distinct basal and luminal cell compartments in which cells express steroid receptors^5^,^7^,^8^,^9^,^10^. In this case, the outer basal layer possesses a discontinuous cellular structure, while the expression of steroid receptors and cell proliferation are strongly decreased, with organoids maintained at most for 14–21 days in culture^5^,^8^,^9^,^10^,^11^,^12^.

In an effort to extend the time over which stem cell activity, functional differentiation and cellular organization can be concurrently maintained within mammary organoids, we identify novel culture conditions, including Neuregulin1 (Nrg1) and low concentrations of R-spondin 1. Importantly, luminal cells retain functional steroid hormone receptor-positive and -negative cells, while basal cells contain functional stem/progenitor cells and differentiated myoepithelial cells, for 2.5 months in culture. We confirm the role of Wnt signalling in driving organoid growth using small molecule Wnt inhibitors and a Tet-O-ΔN89 β-catenin transgenic system. Similarly, lentiviral knockdown of Nrg1 receptors validates the important role of Nrg1 signalling in mammary organoid development. Interestingly, cells capable of forming mammary organoids containing each cell type are identified within the luminal oestrogen receptor (ER)-negative cell population.

## Results

### Nrg1 mediated extended growth of mammary organoids

3D culture conditions that allow the long-term maintenance of several epithelial tissues *in vitro*, including intestine, stomach, liver and prostate, have been recently defined^13^,^14^,^15^,^16^. Here, the basal culture medium (DMEM/F-12 supplemented with penicillin/streptomycin, 10 mM HEPES, Glutamax, 1 × N2, 1 × B27) described in these papers was adapted to include progesterone-free B27 and N2 reagents based on the original formulation from Brewer *et al*.^17^ and Chen *et al*.^18^ (Supplementary Tables 1 and 2).

One of the key constituents of the culture medium enabling the growth of organoids from gastrointestinal and prostate tissues was EGF^13^,^14^,^15^,^16^. Although EGF has been used for decades in multiple mammary gland culture systems^19^, long-term maintenance of both proliferation and functional differentiation of mammary cells *ex vivo* using this factor has not been possible. In an attempt to overcome this limitation, we explored the function of another member of the EGF family of ligands, Nrg1. Nrg1 contains an EGF-like domain that signals by stimulating ErbB receptor tyrosine kinases (ErbB3 and ErbB4) (ref. 20), and is known to regulate different aspects of mammary gland development^21^,^22^,^23^. Mammary epithelial organoids were isolated^24^ and embedded in growth-factor reduced matrigel, a mouse tumour-derived matrix that allows the development of complex 3D structures^13^. Following matrigel polymerization, basal culture medium supplemented with Nrg1 (100 ng ml^−1^) and Noggin (100 ng ml^−1^) was overlaid.

**Figure 1:**
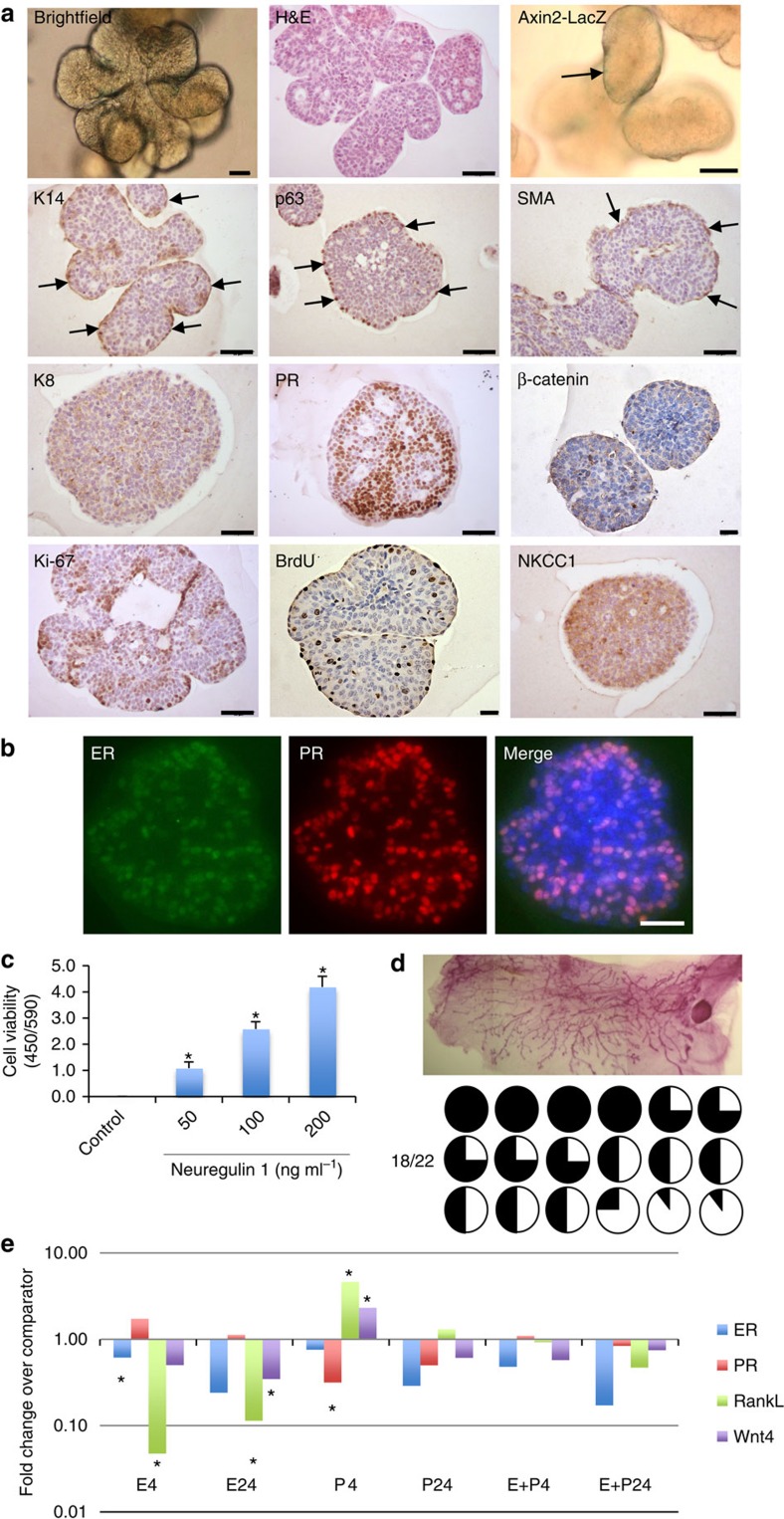
Nrg1 mediates the extended growth of mammary organoids that retain a bilayered organization and response to steroid hormones. (**a**) Mammary epithelial cells were embedded in growth-factor reduced matrigel and cultured in basal culture medium supplemented with Noggin (100 ng ml^−1^) and Nrg1 (100 ng ml^−1^; *n*=5). After 30 days in culture, organoids were fixed, embedded in paraffin and sectioned. Sections were stained for hematoxylin and eosin (H&E), basal markers: K14, p63, SMA; luminal markers: K8, PR; cell-proliferation markers: Ki-67 and BrdU; and cell organization markers: β-catenin and NKCC1. Scale bars, 50 μm, except for bright field, β-catenin and BrdU, 25 μm. Mammary organoids established from Axin2-LacZ (Wnt reporter) mammary glands were fixed and stained for β-galactosidase. (**b**) Detection of ER (green) and PR (red) in mammary organoid section (*n*=5). Counterstain, DAPI (blue). Scale bar, 50 μm. (**c**) Mammary cells were exposed to increasing concentrations of Nrg1. After 30 days in culture, the number of viable cells (Wst assay) was measured (*n*=3, means±s.e.m.). (**d**) Representative picture of mammary gland successfully filled with a ductal tree. Fifty mammary structures cultured for 30 days were transplanted into mammary fat pads of 3-week-old Rag-1 or FvB mice from which the endogenous epithelium was surgically removed. In all, 18 out of 22 mammary fat pads were re-populated with various degrees (from 5 to 100%). (**e**) Expression of *ER*, *PR*, *RankL* and *Wnt4* following treatment with progesterone (P, 40 ng ml^−1^), oestrogen (E, 4 ng ml^−1^) or both hormones. Mammary organoids cultured for 30 days were treated for 4 and 24 h. Gene expression was evaluated by quantitative RT-PCR. Data are expressed as fold change over untreated controls (*n*=3, means±s.e.m.). *P<0.05, paired Student's *t*-test.

Compared with cultures treated with EGF (100 ng ml^−1^) and Noggin (100 ng ml^−1^), Nrg1-treated organoids after 15 days exhibited over a threefold increase in cell viability (Supplementary Fig. 1a), and appeared larger in size (Supplementary Fig. 1b), indicating this ligand to be important in promoting mammary development. By 30 days in culture, these conditions had enabled the growth of complex lobular mammary structures possessing multiple lumens per lobule (Fig. 1a). Organoids were arranged into two distinct cell compartments: an outer layer of cells expressing basal markers including keratin-14 (K14), p63 and smooth muscle actin (SMA), and an inner cell population expressing luminal markers such as keratin-8 (K8), progesterone receptor (PR) and ER (Fig. 1a and Supplementary Fig. 2). Whilst the large majority of basal-like cells co-expressed K14, p63 and SMA, a few basally located, p63-positive cells per structure did not express SMA (Supplementary Fig. 2, white arrows). Mammary cells within the luminal compartment co-expressed ER and PR (Fig. 1b). When cells from Axin2-lacZ knock-in reporter mice were analysed, occasional LacZ-positive cells were observed within the basal cell layers adjoining the matrix, while β-catenin protein was predominantly localized at cell junctions (Fig. 1a and Supplementary Fig. 3a). Interestingly, both basal and luminal cells expressed Na-K-Cl cotransporter 1 (NKCC1), a marker particularly associated with ductal, rather than alveolar, morphology^25^.

Both basal and luminal cells within the organoids were actively cycling based on Ki-67 staining and on the nuclear incorporation of BrdU that had been added 2.5 h before fixation (Fig. 1a). A Nrg1 titration demonstrated that organoid viability and growth were strongly dependent on the addition of Nrg1, reflected by the number and size of organoids formed (Fig. 1c and Supplementary Fig. 4). The specificity of the antibodies used in this study was validated on mammary gland tissue sections (Supplementary Fig. 5), and mouse and rabbit IgG isotype antibodies used as controls (Supplementary Fig. 6).

To demonstrate the maintenance of regenerative potential of these organoids *in vivo* after 30 days in culture, 50 mammary structures were transplanted into the mammary fat pads of 3-week-old mice from which the endogenous epithelium was surgically removed. Most of the host fat pads (18 out of 22) were successfully re-populated after 8 weeks, with the percentage of mammary fat pad filled ranging from 5 to 100% (Fig. 1d).

To determine the functionality of the identified hormone receptors at 30 days in culture, progesterone- and oestrogen-free culture medium was supplemented with progesterone (40 ng ml^−1^) and/or oestrogen (4 ng ml^−1^) for 4 or 24 h. These concentrations are in line with what is previously reported in other culture systems in the literature^11^,^26^,^27^,^28^,^29^. The mRNA expression of *RankL*, *Wnt4*, *PR* and *ER* was then analysed by quantitative PCR. RANKL and Wnt4 have previously been described as paracrine effectors of progesterone *in vivo*^30^. As expected, a 4-h progesterone treatment significantly increased the expression of *RankL* and *Wnt4* (Fig. 1e). Interestingly, *RankL* expression was strongly reduced 4 h after oestrogen treatment, remaining so at 24 h, and exhibited a similar pattern of regulation to that of *Wnt4* (Fig. 1e). Steroid hormone receptor expression is also known to be regulated by steroid hormone treatment^31^. As such, 4 h of oestrogen and progesterone treatment significantly reduced *ER* and *PR* expression respectively. With the exception of *ER* expression, the responses to hormone treatment at 24 h were reduced by comparison with responses at 4 h. Co-treatment of mammary organoids with both hormones had no significant effect on gene expression.

Using a trypsin-based dissociation approach, mammary organoids were maintained for 2.5 months under these conditions, retaining distinct cellular organization with functional steroid hormone receptors and normal karyotype (Supplementary Fig. 7). As previously described^15^,^16^,^32^, chromosomal spreads with 40±1 chromosomes were considered normal, due to the assay limitations.

Taken together, these results suggest that Nrg1 may be a critical regulator of mammary cells, that *in vitro* promotes the development of mammary organoids with differentiated basal and luminal cell compartments, functional steroid hormone receptor-positive and -negative luminal cells and stem/progenitor cells. Given this observation, we investigated whether Nrg1 has a biological relevance in the mammary gland *in vivo,* by first determining the expression levels of the growth factor and its receptors in different mammary epithelial cell populations.

### Nrg1 and its receptors show reciprocal expression

**Figure 2:**
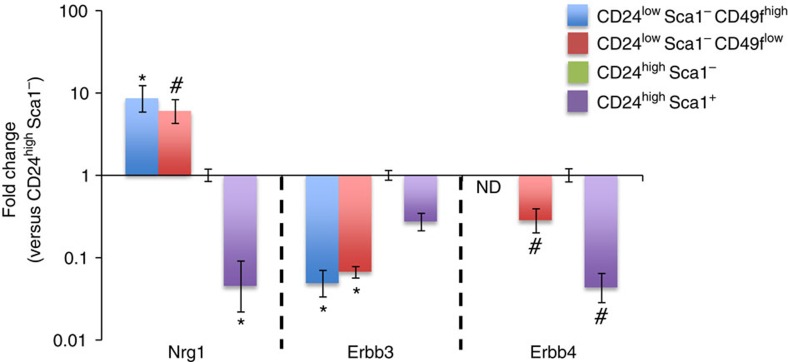
Nrg1 and ErbB are expressed in distinct mammary cell populations. Mammary epithelial cell populations were FACS sorted using a panel of antibodies: CD24^low^ Sca-1^−^ CD49f^high^ (stem cell-enriched fraction), CD24^low^ Sca-1^−^ CD49f^low^ (myoepithelial cell population), CD24^high^ Sca-1^−^ (luminal ER-negative cells) and CD24^high^ Sca-1^+^ (luminal ER-positive cells). Expression of *Neuregulin 1*, *Erbb3* and *Erbb4* was evaluated by quantitative RT-PCR (*n*=3 independent experiments). Fold expression±95% confidence over the comparator population (CD24^high^Sca1^−^). ND, no expression of Erbb4 detected. *P<0.05; ^#^0.05<*P*<0.1 (versus CD24^high^ Sca1^−^), unpaired Student's *t-*test.

To define the expression pattern of Nrg1 and its receptors in the mammary gland, mammary epithelial cell populations (luminal ER^+^, luminal ER−, myoepithelial and stem cell-enriched) from 10-week-old FvB mice were FACS sorted using the previously described lineage markers CD24, Sca1 and CD49f (refs 33, 34). mRNA expression levels of *Nrg1*, *Erbb3* and *Erbb4* were investigated by qRT-PCR (Fig. 2). *Nrg1* and its receptors were expressed in luminal CD24^high^ Sca1^+^ (ER^+^) and CD24^high^ Sca1^−^ (ER^−^) cell populations and in the basal myoepithelial CD24^low^ Sca1^−^ CD49f^low^ and stem cell-enriched CD24^low^ Sca1^−^ CD49f^high^ cell populations, but *Erbb4* was not detected in the CD24^low^ Sca1^−^ CD49f^high^ population. Expression of *Nrg1* was higher in basal cells than luminal cells, as recently described^35^, while the Nrg1 receptors *Erbb3* was expressed at higher levels in luminal cells than in myoepithelial and stem cell-enriched fractions. *Erbb4* was expressed at higher levels in CD24^high^ Sca1^−^ cells. This result suggests the potential for a paracrine signalling network whereby basal cells secrete Nrg1, which acts on luminal cells *via* Erbb3 and Erbb4 receptors.

### Establishment of mammary cultures that resemble Wnt1 tumours

Wnt signalling plays a critical role in mammary gland development, from regulating the elongation of the ductal tree-like structure to pregnancy-induced lobulo-alveolar development^36^. Due to similarities in Wnt signalling requirements for stem cell function in the small intestine and mammary gland, the growth factors that are required to maintain intestinal organoids, namely EGF, Noggin and R-spondin 1 (Rspo), were tested in our system^13^. In intestinal stem cells, Rspo potentiates Wnt signalling, functioning as a ligand for leucine-rich repeat containing G-protein-coupled receptor 5 (Lgr5), which forms a complex with Wnt coreceptors^37^.

**Figure 3:**
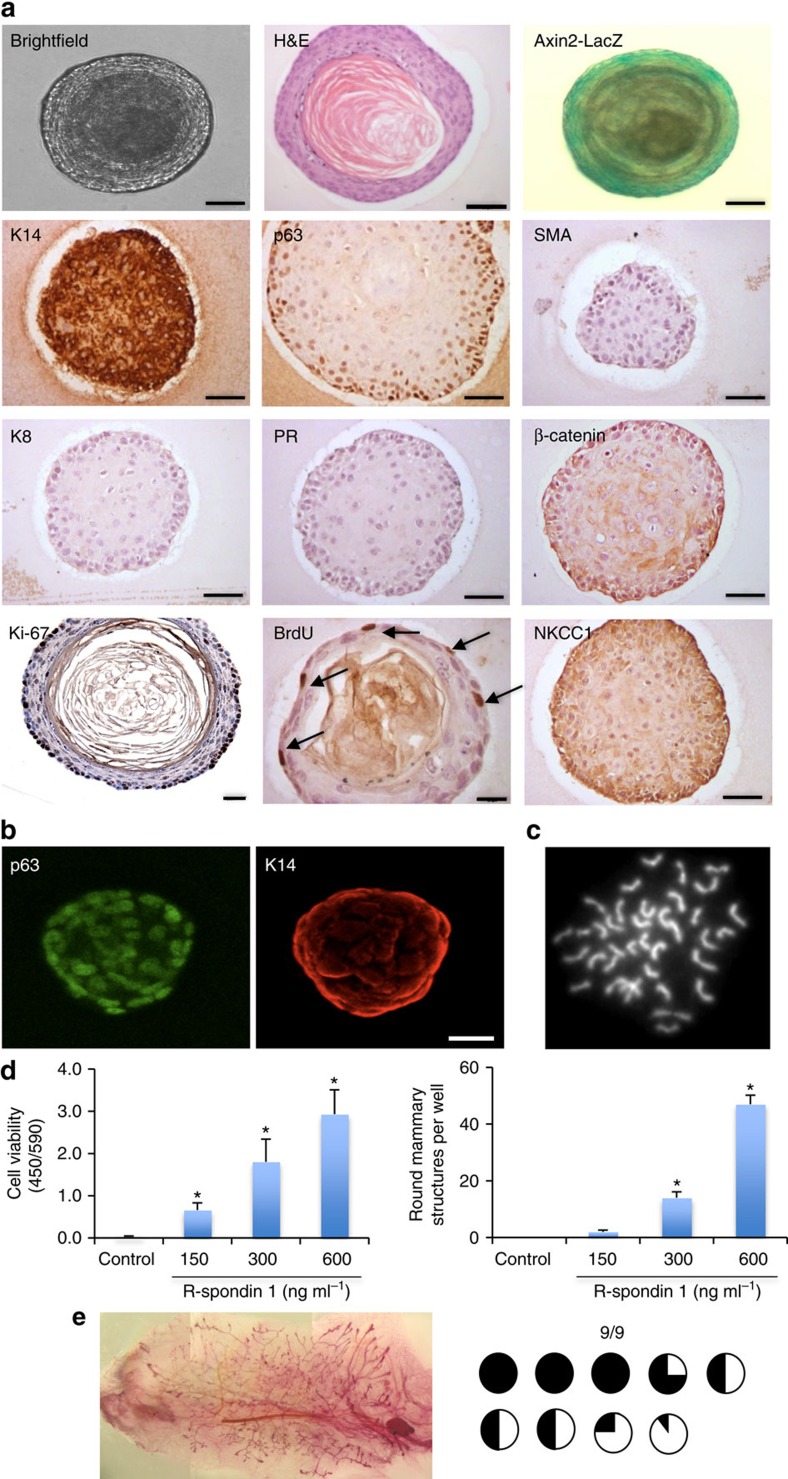
R-spondin 1 promotes the growth of mammary organoids that are enriched in basal cells. (**a**) Mammary epithelial cells (*n*=5) were embedded in growth-factor reduced matrigel. After polymerization of matrigel, basal culture medium supplemented with R-spondin 1 (600 ng ml^−1^), Noggin (100 ng ml^−1^) and EGF (50 ng ml^−1^) was overlaid. After 18 days in culture, organoids were fixed, embedded in paraffin and sectioned. Sections were stained for hematoxylin and eosin (H&E), basal markers: K14, p63, SMA; luminal markers: K8, progesterone receptor (PR); cell-proliferation markers: Ki-67 and BrdU; and cell organization markers: β-catenin and NKCC1. Scale bars, 50 μm, 30 μm (BrdU) and 25 μm (Ki-67). Mammary organoids established from Axin2-LacZ (Wnt reporter) mammary glands were fixed and stained for β-galactosidase. (**b**) Confocal images of organoids stained for p63 (green) and K14 (red; *n*=5). Scale bar, 50 μm. (**c**) Representative picture of metaphase chromosome spread that shows a normal number of chromosomes. (**d**) Mammary cells were exposed to increasing concentrations of R-spondin 1. After 18 days in culture, cell viability (Wst assay) and the number of structures were evaluated (*n*=3, means±s.e.m.). (**e**) Representative picture of mammary gland successfully filled with a ductal tree. Mammary structures cultured for 30 days were transplanted into mammary fat pads of 3-week-old FvB mice from which the endogenous epithelium was surgically removed. In all, 9 out of 9 mammary fat pads were re-populated with various degrees (from 5 to 100%). *P<0.05, paired Student's *t*-test.

Mammary epithelial organoids were isolated and embedded as previously described, and overlaid with basal culture medium supplemented with Rspo (600 ng ml^−1^), Noggin (100 ng ml^−1^) and EGF (50 ng ml^−1^). After 18 days in culture under these conditions, mammary epithelial organoids predominantly form large oval or round structures (150–300 μm) that organize around a central lumen (Fig. 3a). The epithelial cells express basal cell markers, including p63 and K14, but the expression of SMA and the luminal markers K8 and PR was undetectable (Fig. 3a,b). Activation of Wnt signalling driven by Rspo here resembled the formation of basal-enriched organoids previously reported following inhibition of Notch signalling in mammary cell culture^38^. The epithelial cells, comprising a p63^+^ K14^+^ basal-like population, were actively proliferating (Ki-67^+^ and BrdU^+^) and the majority of cells were Wnt responsive (Axin2-LacZ^+^; Fig. 3a). However, the nuclear accumulation of β-catenin, a marker of Wnt signalling activation, was not observed, with its localization instead detected at cell junctions (Supplementary Fig. 3b). The cultured mammary cells expressed NKCC1 (Fig. 3a), suggesting a ductal epithelial status of the cells in culture. This phenotype was maintained to 30 days in culture, while the karyotype at this point was essentially normal (71% of analysed cells contained 40±1 chromosomes, *n*=7; Fig. 3c).

Cell viability and the proportion of mammary cells and structures was also dependent on the absolute levels of Rspo. In the absence of Rspo, only small spherical structures that could not be maintained for long term in culture were observed (Fig. 3d and Supplementary Fig. 8a). Low Rspo levels maintained slow organoid growth while high concentrations supported strong cell proliferation and formation of basal-like structures (Fig. 3d and Supplementary Fig. 8a). The activation of Wnt signalling in this system was largely Rspo dependent, as removal of Rspo markedly reduced the number of Axin2-LacZ-positive mammary structures (Supplementary Fig. 8b,c).

The core of these non-transformed mammary structures resembled typical squamous metaplasia observed in mammary tumour tissues^39^, including MMTV-Wnt1-driven mammary tumours that express K14 and p63 basal markers (Supplementary Fig. 9).

To assess the regenerative potential of these organoids at 30 days in culture, fifty basal cell-enriched organoids were transplanted into cleared mammary fat pads of 3-week-old mice, and all host mammary fat pads (9/9) were successfully filled with a complete ductal tree after 8 weeks (Fig. 3e). Using a trypsin-based dissociation approach, these mammary organoids and their associated phenotype were maintained in culture long-term (3.5–5 months), but exhibited an increase in chromosomal number by this point (Supplementary Figs 10 and 11).

Taken together, the Rspo supplementation studies suggest that Wnt pathway activity may promote organoid viability at low levels, while driving basal differentiation that recapitulates Wnt1-driven oncogenesis *in vivo* at high levels.

### Nrg1 and Rspo support mammary organoid development *in vitro*

Having established independently the importance of Nrg1 and Wnt activation in promoting mammary organoid culture, in an effort to maintain both luminal and basal cell compartments *in vitro* the Nrg1 culture medium was supplemented with a low concentration of Rspo (100 ng ml^−1^).

**Figure 4:**
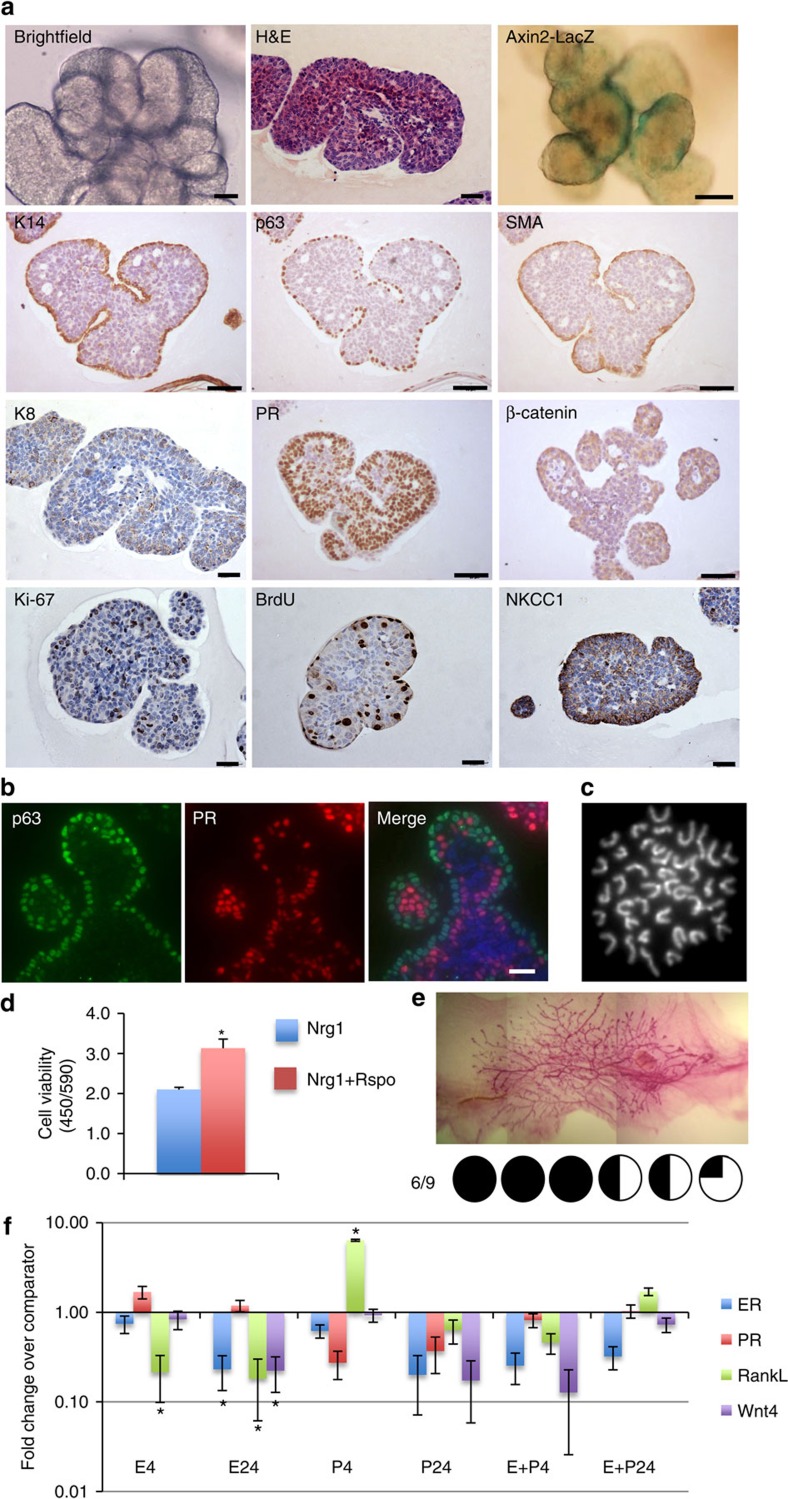
Nrg1 and R-spondin 1 enhance the formation of a basal cell layer while maintaining the luminal compartment. (**a**) Mammary epithelial cells were embedded in growth-factor reduced matrigel. After polymerization of matrigel, basal culture medium supplemented with Noggin (100 ng ml^−1^), R-spondin 1 (100 ng ml^−1^) and Nrg1 (100 ng ml^−1^) was overlaid. After 30 days in culture, organoids were fixed, embedded in paraffin and sectioned (*n*=5). Sections were stained for hematoxylin and eosin (H&E), basal markers: K14, p63, SMA; luminal markers: K8, PR; cell-proliferation markers: Ki-67 and BrdU; and cell-organization markers: β-catenin and NKCC1. Scale bars, 50 μm except for bright field, H&E, K8, Ki-67, BrdU and NKCC1 (25 μm). Mammary organoids established from Axin2-LacZ (Wnt reporter) mammary glands were fixed and stained for β-galactosidase. (**b**) Detection of p63 (green) and PR (red) in mammary organoid section. Counterstain, DAPI (blue; *n*=5). Scale bar, 50 μm. (**c**) Representative picture of metaphase chromosome spread that shows a normal number of chromosomes. (**d**) Mammary cells were treated with Nrg1 (100 ng ml^−1^) or Nrg1+R-spondin 1 (100 ng ml^−1^ each) for 30 days. The number of viable cells (Wst assay) was evaluated (*n*=3, means±s.e.m.). (**e**) Representative picture of mammary gland successfully filled with a ductal tree. Mammary structures cultured for 30 days were transplanted into mammary fat pads of 3-week-old FvB mice from which the endogenous epithelium was surgically removed. In all, 6 out of 9 mammary fat pads were re-populated with various degrees (from 15 to 100%). (**f**) Expression of *ER*, *PR*, *RankL* and *Wnt4* following treatment with progesterone (P, 40 ng ml^−1^), oestrogen (E, 4 ng ml^−1^) or both hormones. Mammary organoids cultured for 30 days were treated for 4 and 24 h and gene expression evaluated by quantitative RT-PCR. Data are expressed as fold change (versus untreated, *n*=3, means±s.e.m.). *P<0.05, paired Student's *t*-test.

After 30 days in culture, mammary organoids formed lobular structures containing multiple lumens per lobule (Fig. 4a). In contrast to Nrg1-only conditions, Axin2-LacZ positivity was more strongly detected, and located at the edge of the mammary structures where the K14^+^/p63^+^/SMA^+^ basal layer resided (Fig. 4a and Supplementary Figs 12 and 13a), in line with observations *in vivo* where Wnt-responsive cells have been demonstrated to be located in the basal layer of mature ducts^6^. In addition, in accordance with these findings, treatment of fully grown mammary organoids with 100 ng ml^−1^ Rspo for 6 days promoted the activation of Wnt signalling, as marked by the presence of Axin2-LacZ-positive cells at the edge of mammary organoids (Supplementary Fig. 13b), although nuclear accumulation of β-catenin was not observed (Supplementary Fig. 3c). When compared with Nrg1-only conditions, the basal cell layer was continuous, with no gaps between basal cells observed (Fig. 4a,b and Supplementary Fig. 12b). Crucially, in contrast to organoids grown under EGF, Rspo-high conditions, structures under Nrg1, Rspo-low did not show any hyperkeratinisation. At 30 days, these mammary organoids were karyotypically normal (Fig. 4c and Supplementary Fig. 12a). Cell numbers as assessed by mitochondrial activity were increased by 50% following the addition of Rspo and Nrg1 (Fig. 4d). Time lapse imaging over 4 days suggested that this was due to increased organoid size (Supplementary Movies 1 and 2).

Mammary structures grown in Nrg1-Rspo exhibited differentiation and maintenance of a K8-positive luminal cell population co-expressing ER and PR (Fig. 4a and Supplementary Fig. 12) for at least 30 days in culture. Importantly, following treatment with EdU 2 h before fixation, co-immunofluorescence analysis revealed that PR-positive cells were distinct from EdU-positive proliferating cells (Supplementary Fig. 14), as observed in the normal mature duct *in vivo*^40^. Organized basal and luminal compartments were clearly distinguishable, as p63-positive cells and PR-positive cells were not co-localized (Fig. 4b). Early time points were also assessed, highlighting this specific cellular organization to already be present in culture at day 3.5 and maintained at days 7 and 14 (Supplementary Fig. 15). Within the basal compartment, rare p63-positive SMA-negative cells were detected, corroborating observations *in vivo* where a small number of K14^+^/SMA- cells were identified within sorted basal myoepithelial populations^34^ (Supplementary Fig. 12, white arrows), although the function of this rare population remains unclear. NKCC1 and β-catenin was localized at the cell membrane of both basal and luminal cells at 30 days in culture (Fig. 4a). Notably, Nrg1 was an absolute requirement as low-dose Rspo treatment alone failed to generate mammary structures (Supplementary Fig. 16).

Nrg1/Rspo-dependent organoids successfully re-populated cleared mammary fat pads (6 out of 9 fat pads) on transplantation (Fig. 4e). Furthermore, as observed in Nrg1-only culture conditions, oestrogen and progesterone had opposing effects on *RankL* gene-expression levels in organoids grown under these conditions (Fig. 4f). Similarly, oestrogen has been reported to result in depression of *RankL* mRNA *in vivo*^41^, and may therefore inhibit progesterone-induced *RankL* expression. Mammary organoids containing distinct luminal and basal compartments were also successfully generated from C57BL/6 mammary cells (Supplementary Fig. 17).

**Figure 5:**
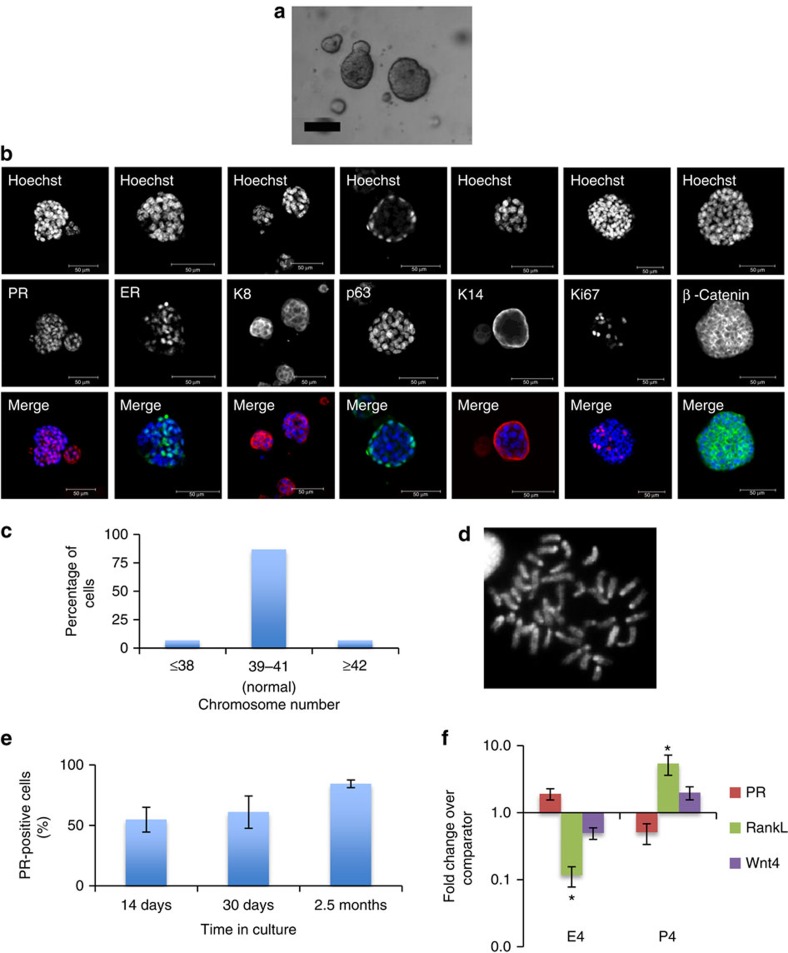
Organoids grown for 70 days under Nrg1/Rspo culture conditions retain normal phenotype and functional steroid receptors. Mammary organoids were cultured with 2.6 ng ml^−1^ R-spondin 1 (Peprotech), 100 ng ml^−1^ Nrg1 and 100 ng ml^−1^ Noggin for 70 days (4 passages). (**a**) Representative bright field image of the organoids. Scale bar, 100 μm. (**b**) Organoids were fixed and stained for luminal markers PR, K8 and ER, basal markers K14, p63, and Ki-67 and β-catenin. Counterstain, hoechst (blue). Scale bars, 50 μm. (**c**) Quantification of chromosome number in mammary organoids cultured for 70 days (*n*=15). Chromosomal spreads with 40±1 chromosomes were considered normal. (**d**) Representative picture of metaphase chromosome spread that shows a normal number of chromosomes. (**e**) Percentage of PR-positive cells per organoid at different time points in culture (*n*=3). (**f**) Expression of *PR*, *RankL* and *Wnt4* following treatment with oestrogen (E, 4 ng ml^−1^) or progesterone (P, 40 ng ml^−1^) for 4 h. The gene expression was evaluated by quantitative RT-PCR. Data are expressed as fold change (versus untreated, *n*=3, means±s.e.m.). *P<0.05, paired Student's *t*-test.

**Figure 6:**
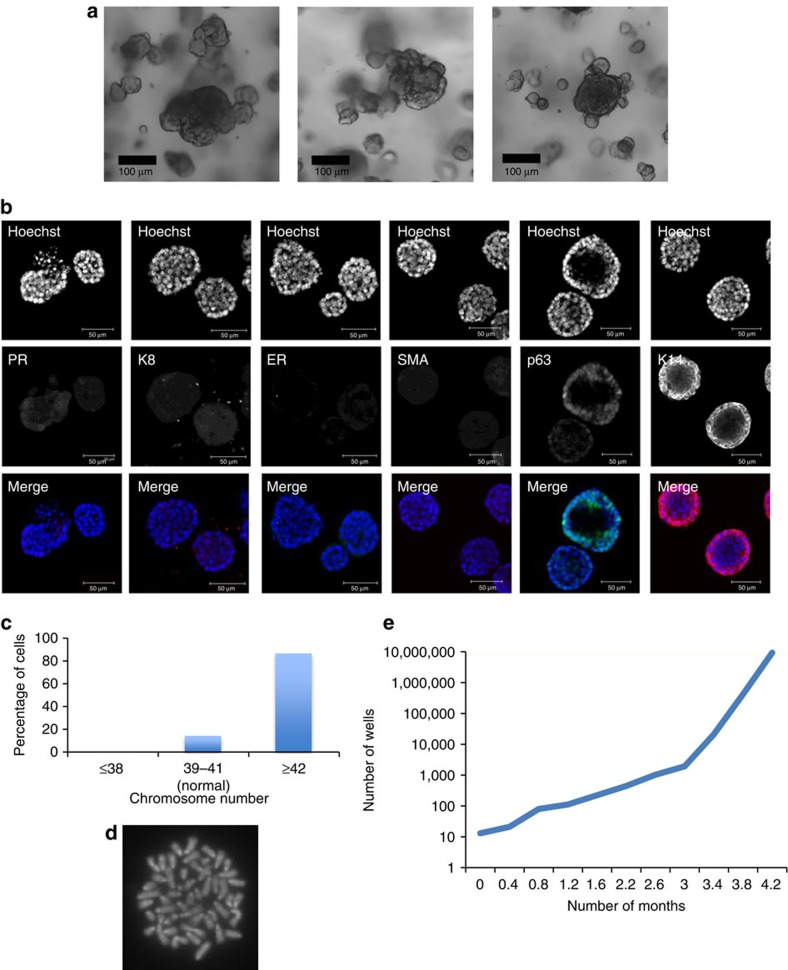
Long-term culture of mammary organoids with Nrg1 and R-spondin 1 promotes chromosomal abnormalities. Mammary epithelial cells were freshly isolated, embedded in matrigel, exposed to culture medium containing Nrg1 (100 ng ml^−1^), Noggin (100 ng ml^−1^) and R-spondin 1 (2.7 ng ml^−1^), and cultured for 112 days (passaged 10 times). (**a**) Representative bright-field images of mammary organoids. Scale bar, 100 μm. (**b**) Mammary organoids were fixed and stained for the basal markers SMA, p63 and K14, and luminal markers PR, K8 and ER (DAPI, blue). Scale bar, 50 μm. (**c**) Quantification of chromosome number in mammary organoids (*n*=29). Chromosomal spreads with 40±1 chromosomes were considered normal. (**d**) Representative picture of metaphase chromosome spread that shows an aberrant number of chromosomes. (**e**) Quantification of organoid expansion over time.

Using a trypsin-based dissociation approach, karyotypically normal, functional mammary organoids and their associated phenotype were able to be maintained in extended culture for 2.5 months (Fig. 5 and Supplementary Table 3). In addition, over this period of time, the number of organoids was 100-fold increased compared with day 0 (Fig. 6e). However, after 4 months in culture, cells exhibited an increase in chromosomal number, an alteration of cell architecture with loss of luminal cells and increased proliferative capacities (Fig. 6), indicative of aberrant changes in organoid cultures at this time point.

To further investigate the requirement of the mammary organoids for Wnt activity, an inducible Tet-O-ΔN89β-catenin mouse model was used^42^. This system allows doxycycline-dependent expression of activated β-catenin. Organoids were grown under Nrg1-only conditions, supplemented with either low concentrations of Rspo, doxycycline or both in combination. Results confirmed that a low level of Wnt signalling induced ‘normal' organoid morphology, while constitutive β-catenin expression induced a squamous-like organoid morphology resembling MMTV-Wnt1 tumours (Supplementary Fig. 18). Furthermore, inhibitors of the Wnt pathway (IWP-2; Wnt secretion inhibitor and IWR-1; Tankyrase inhibitor) reduced organoid growth and PR expression, suggesting that mammary organoids and hormone receptor-positive luminal cells in particular have a basic requirement for Wnt ligand activity (Supplementary Figs 19–22). Similarly, to characterize the requirement of the mammary organoids for Nrg1/ErbBs active signalling, a shRNA-mediated knock down of *ErbB3* and *ErbB4* was applied. Freshly isolated mammary epithelial cells were transduced with lentiviral vectors and the size of the resulting transduced (GFP^+^) organoids was measured compared with that of a scrambled control, after 10 days in culture. Results illustrated a reduction in organoid area on knockdown of either receptor, with *ErbB4* knockdown in particular restricting organoid size to only 60% of control (*P*<0.01; Supplementary Fig. 23). In addition, organoids grown under Nrg1-Rspo conditions were successfully used to demonstrate an additional growth-promoting effect of hepatocyte growth factor (HGF) without altering organoid marker expression (Supplementary Figs 24–26).

### Luminal ER-negative cells provide all cell lineages *in vitro*

**Figure 7:**
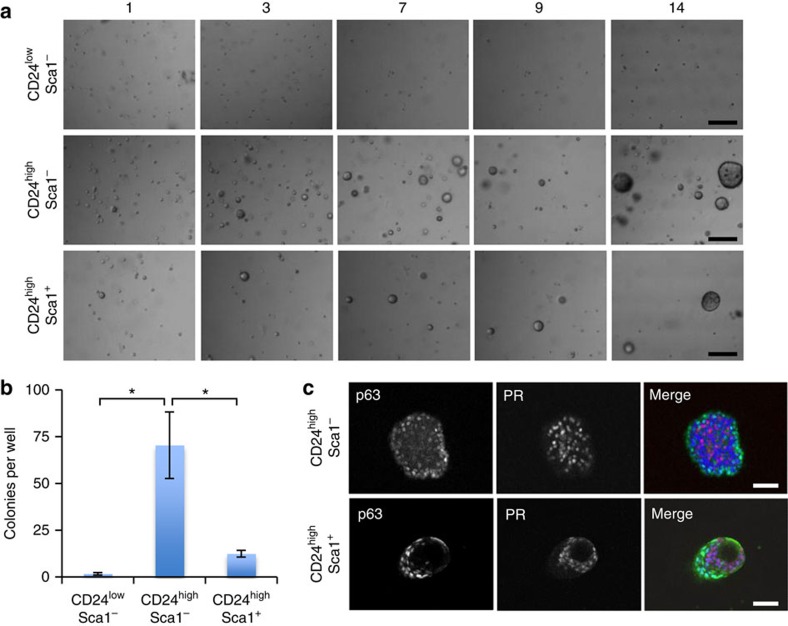
Mammary organoid-forming cells are located in the luminal ER-negative cell population and provide all the cell lineages *in vitro*. (**a**) Representative pictures of mammary organoids originating from single CD24^high^ Sca-1^−^ cells (luminal ER-negative fraction), CD24^high^ Sca-1^+^ cells (luminal ER-positive fraction) or single basal cells (CD24^low^). Scale bars, 100 μm. (**b**) Colony-forming efficiency from the three different cell populations (*n*=3, means±s.e.m.). (**c**) CD24^high^ Sca1^−^ and CD24^high^ Sca-1^+^ cells generate mammary organoids that contain distinct luminal and basal compartments (*n*=3). Scale bars, 50 μm. *P<0.05, paired Student's *t*-test.

To investigate the cell of origin of the mammary organoids, single FACS-sorted mammary cells of 10-week-old virgin mice were embedded in matrigel and overlaid with medium containing Nrg1 (100 ng ml^−1^), Rspo (100 ng ml^−1^) and the Rho Kinase inhibitor Y-27632 (10 μM), previously demonstrated to improve cell survival following cell sorting^13^. Mammary organoids arose predominantly from the CD24^high^ luminal sub-populations and were rarely observed in the CD24^low^ basal sub-population (Fig. 7a). The colony-forming efficiency of CD24^high^ Sca1^−^ luminal cells was nearly 6 and 40 times higher than those of CD24^high^ Sca1^+^ luminal cells and CD24^low^ Sca1^−^ basal cells, respectively (Fig. 7b). The mammary structures that formed from the CD24^high^ Sca1^−^ luminal ER^−^ cell population contained distinct luminal and basal cell compartments, as marked by the presence of an outer layer of p63/K14/K5-positive cells and an inner population of ER/PR/K8-positive cells (Fig. 7c and Supplementary Fig. 27). Similar results were obtained for the CD24^high^ Sca1^+^ luminal ER^+^ cells (Fig. 7c). Due to the low frequency of basal outgrowths, their in depth analysis was not possible. Collectively, these results suggest that the luminal population, predominantly the ER^−^ mammary epithelial cells, contain progenitor cells that can, under the conditions defined, directly contribute to the formation of both luminal and basal compartments, as we initially proposed^43^.

## Discussion

Recent advances in 3D culture systems have enabled the establishment of organoids from various tissues that are remarkably similar in organization and structure to the organs that they are derived from^4^. Here, we describe the first defined *in vitro* culture system that robustly recapitulates the murine mammary duct differentiation found *in situ* and allows the extended maintenance of normal primary mammary epithelial cells. Rspo/Nrg1-dependent mammary organoids contain distinct basal and luminal cell compartments, the latter comprising of both ER^+^ and ER^−^ subtypes and progenitor cells that enable organoid expansion for multiple passages over the course of 2.5 months, a significant advancement over previously described mammary culture conditions^5^,^8^,^9^,^10^,^11^,^12^. Crucially, the utility of the system is further enhanced by the maintenance of hormone responsive cells that retain functional steroid receptors capable of regulating known downstream target genes, such as *RankL* and *Wnt4*. Furthermore, the organized 3D structures grown here also have full regenerative potential as demonstrated by their ability to successfully recreate a complete mammary ductal tree *in vivo*. Moreover, organoids can be genetically manipulated in culture for further study or transplantation. Altogether, the data suggest that this *in vitro* 3D culture system can serve as an instrumental tool for investigating mammary gland biology, in addition for potential drug-screening applications, as demonstrated here using Wnt signalling inhibitors and HGF supplementation.

The reproductive history and lifetime hormone exposure of a woman is intimately linked to her breast cancer risk^44^. As such, a further understanding of normal mammary gland development, in particular the molecular mechanisms of hormone action, is critical for identifying the events that can lead to breast tumourigenesis. Steroid hormones have been reported to expand mammary stem cells *in vivo*, although the precise factors that contribute towards maintaining the stem cell niche remain poorly defined^30^,^45^. Although a recent study has demonstrated maintenance of PR expression in culture over 14 days^10^, most previous culture systems have failed to maintain long-term expression of *ER* and *PR* in 3D cultures of normal mammary epithelium, leaving the investigation of mechanisms of hormonal regulation to be principally driven by the use of ER/PR^+^ breast cancer cell lines *in vitro*. As such, the mammary 3D organoid culture method described provides an excellent system in which to study such regulation of normal mammary epithelial cells. Notably, a recent study utilizing a highly similar matrigel-based 3D *in vitro* system demonstrated that single luminal epithelial progenitors can generate prostate organoids in culture, and that these also retained functional hormone (androgen) receptor expression and downstream signalling^46^.

**Figure 8:**
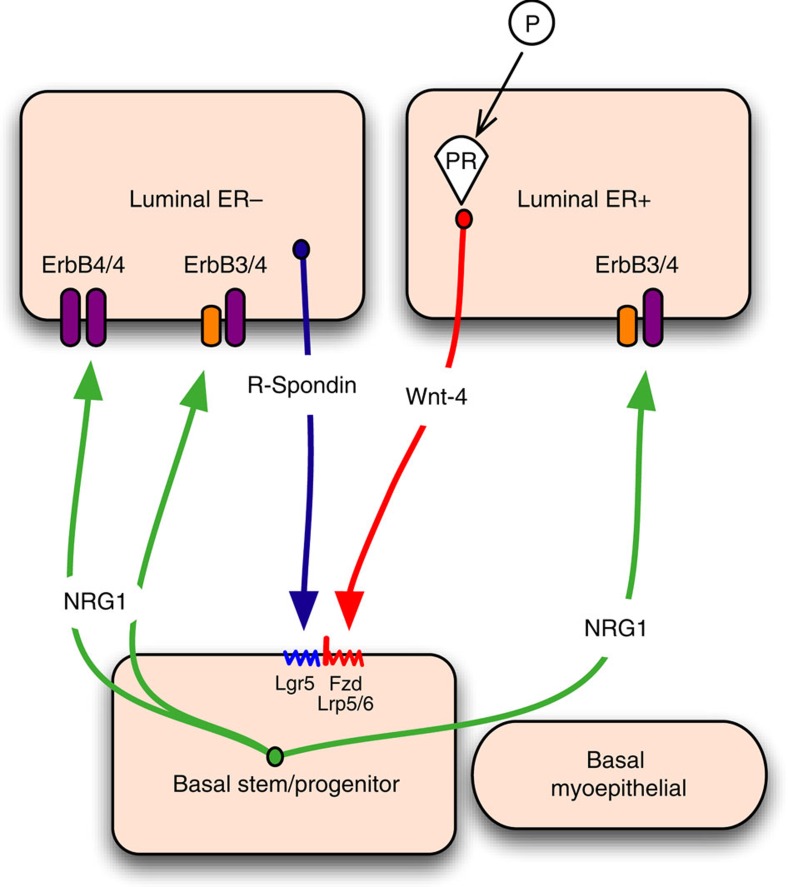
Model of the Wnt/Nrg1 paracrine signalling network between luminal and basal mammary epithelial cells. Basal cells secrete Nrg1, resulting in Nrg1/ErbB signalling activation in luminal cells, promoting luminal progenitor cell expansion and differentiation. In contrast, progesterone-stimulated Wnt4 secretion by luminal ER^+^ cells, alongside R-spondin 1 produced by luminal ER^−^ cells, activate Wnt signalling in the basal stem/progenitor cell population via the Wnt receptors Fz and Lrp5/6 and the R-spondin 1 receptor Lgr5. Wnt signalling activation in this compartment is likely to promote growth and differentiation of the basal cell layer. Fz, frizzled; Lgr5, leucine-rich repeat-containing G-protein-coupled receptor 5; Lrp5/6, low-density lipoprotein receptor-related protein 5/6.

The Wnt signalling pathway is an important regulator of ductal and lobulo-alveolar development and contributes to the self-renewal and maintenance of basal cells, including mammary stem cells^6^,^36^. The Wnt signalling potentiator Rspo has been implicated in many *in vitro* systems that maintain and expand adult tissue stem cells, including the intestine^13^, stomach^14^ and liver^15^. The presence of Rspo was required in our (Neu/Rspo-low) culture system, to support the growth and differentiation of the basal cell layer. Interestingly, a recent study showed that mammary luminal ER/PR-negative cells selectively secrete Rspo, while *Wnt4* is predominantly expressed in ER/PR-positive cells^47^, suggesting a model for the hormonal regulation of basal mammary stem cells, whereby these two luminal cell types provide the niche (Fig. 8). In further support of this, the induction of Wnt4 by progesterone in luminal ER/PR-positive cells has been suggested to drive lobular development in pregnancy via its effects on basal stem cells^47^. However, our studies argue that the highest Rspo levels induce the development of undifferentiated and basal-only structures, together with squamous metaplasia, as characteristic of MMTV-Wnt1-driven mammary tumours *in vivo.* This interesting observation suggests that a ‘just right' level of Wnt signalling may be necessary for normal mammary development in which differentiated luminal and basal cells are present within the same structures^48^, in contrast to other tissues (for example, the intestine), which require high levels of R-spondin 1 for long-term 3D *in vitro* culture. The requirement for basal-to-luminal Nrg1/ErbB4 signalling in pregnancy has also been recently reported^35^. Nrg1, which is reportedly secreted by basal cells, directly regulates ErbB4-positive luminal progenitors and promotes their proliferation and differentiation^35^. The work presented here supports and refines this analysis by demonstrating that cells that respond to Nrg1 are more likely to reside in the population of luminal ER^−^ cells that express high levels of the *ErbB3* and *ErbB4* Nrg1 receptors (Fig. 8). Importantly, we observed that CD24^high^ Sca1^−^ luminal ER^−^ cells generate mammary organoids containing distinct epithelial compartments, suggesting that these cells can contribute to both luminal and basal cell lineages *in vitro*, and highlighting their potential high plasticity. While this finding contrasts with the current model of mammary cell hierarchy whereby luminal progenitors remain committed to the luminal lineage *in vivo*^1^,^2^, in the context of transplantation, it is not uncommon for luminal ER^−^ progenitor cells to generate both basal and luminal cell lineages^29^,^49^, implying that transdifferentiation of these cells may be possible in unusual physiological settings. Indeed, luminal-to-basal reversion has been documented in breast cancer where luminal tumour cells can acquire basal identity during invasion^50^.

Mammary epithelial cells expanded and maintained for 2.5 months under the described culture conditions retain normal cellular architecture and growth potential, and crucially, using karyotype analysis previously implemented in multiple published organoid culture studies^15^,^16^,^32^, a normal (39–41) chromosome count was observed after this period in culture. Intriguingly, unlike comparable *in vitro* systems for the intestine that can maintain karyotypically normal cells in culture for over a year^13^, we observed that mammary organoids acquired chromosomal abnormalities after 4 months. Whilst the intestine is required to continually regenerate throughout life to maintain normal physiological function, the inherent lifespan of the mammary gland in contrast is relatively finite. In agreement with our findings, Daniel and Young^51^ observed that growth senescence was achieved on serial transplantation of mammary epithelial cells, in a manner directly related to the number of cell divisions. Our observation may therefore reflect the intrinsic biology of the breast as compared with other tissues, and merits further investigation in future studies.

By facilitating the simultaneous growth and maintenance of multiple mammary epithelial cell types in culture, the *in vitro* system described herein allows hormonal regulation and the inter-cellular signalling between basal and luminal compartments to be studied in an accessible, but normal mammary epithelial cell environment. Furthermore, organoids are amenable to lentiviral infection thus facilitating their genetic manipulation in culture, in addition to possessing great potential as a drug-screening tool. Finally, this system may facilitate the establishment of cancer organoid culture conditions, given that tumour cells will have arisen within a similar complex and heterogeneous environment.

## Methods

### Animal models and maintenance

All animal work was carried out under UK Home Office project and personal licences following local ethical approval and in accordance with local and national guidelines.

Adult Axin2-LacZ^52^, Tet-O-ΔN89β-catenin^42^, wild-type FvB and C57BL/6 female mice (8–16 weeks old) were used in the experiments. Animals were maintained under a 12/12 light/dark cycle at a temperature of 20 °C with free access to food and water.

### Mammary organoid culture

Mammary epithelial organoids were collected from fourth mammary fat pads of 8–12-week-old virgin mice^24^. Briefly, fat pads were chopped three times with a McIlwain Tissue Chopper (Mickle Laboratory Engineering Company, Surrey, UK) and the finely minced tissue was transferred to a digestion mix consisting of serum-free Leibowitz L15 medium (Gibco) containing 3 mg ml^−1^ collagenase A (Sigma) and 1.5 mg ml^−1^ trypsin (Sigma). This was incubated for 1 h at 37 °C to liberate epithelial tissue fragments (‘organoids'). Isolated organoids were mixed with 50 μl of Matrigel (BD Biosciences) and seeded in 24-well plates. The basal culture medium contained phenol red free DMEM/F-12 with penicillin/streptomycin, 10 mM HEPES, Glutamax, N2 and B27. Progesterone-free B27 and N2 reagents were developed based on the original formulation from Brewer *et al*.^17^ and adapted from Chen *et al*.^18^ (Supplementary Tables 1 and 2)^17^,^18^. The basal medium was supplemented with three different growth factor cocktails: (i) Nrg1 (100 ng ml^−1^, R&D) and Noggin (100 ng ml^−1^, Peprotech), or (ii) EGF (50 ng ml^−1^, Sigma Aldrich), R-spondin 1 (600 ng ml^−1^, R&D, or 42.5 ng ml^−1^ Peprotech) and Noggin (100 ng ml^−1^, Peprotech), or (iii) Nrg1 (100 ng ml^−1^, R&D), R-spondin 1 (100 ng ml^−1^, R&D or 2.656ng ml^−1^ Peprotech) and Noggin (100 ng ml^−1^, Peprotech). Lower concentrations of recombinant R-spondin 1 from Peprotech were used compared with R-spondin 1 from R&D due to its superior activity. In all, 500 μl of supplemented basal culture medium was added per well and organoids were maintained in a 37 °C humidified atmosphere under 5% CO_2_.

Mammary organoids were maintained in culture for several months by passaging them every 2–3 weeks using both mechanical and enzymatic approaches (1:2 or 1:3 split ratio approximately). Briefly, mammary organoids were released from the matrigel by breaking the matrix with a P1000 pipette and were treated with trypsin-EDTA 0.05% for 5 min at 37 °C. Following incubation with trypsin inhibitor for 2 min, mammary cells were strained through a 70 μm cell strainer (BD Biosciences) on ice and centrifugated at 1,500 r.p.m. for 5 min at 4 °C. Mammary cells were then resuspended in matrigel (50,000 cells per 50 μl), seeded in 24-well plates and exposed to previously described culture conditions.

### Mammary organoid cryopreservation

Organoids were grown for 3–4 days to allow small structures to establish, before removal from Matrigel via washing in cold DMEM/F-12 (up to a maximum of 6 wells per 12 ml media), and centrifugation at 1,500 r.p.m. for 5 min. Organoids were then resuspended in freezing media (50% DMEM/F-12, 40% FBS, 10% DMSO) at a density equivalent to 1 well ml^−1^, and aliquoted at 1 ml per cryovial. Cryovials were stored at −80 °C. For thawing, vials were placed in a 37 °C waterbath and the contents washed twice in DMEM/F-12, before reseeding in Matrigel at required density. Thawed organoids were plated for the first 5 days with media supplemented with Y-27632 (10 μM).

### Flow cytometry and cell sorting

Mammary epithelial organoids were collected from fourth mammary fat pads of 10-week-old virgin mice as described above and processed to single cells. Briefly, isolated epithelial fragments were resuspended in serum-free Joklik's Modification of Minimal Essential Medium for Suspension Culture and incubated in a water bath at 37 °C for 15 min. Fragments were digested to single cell using Trypsin-EDTA 0.05% (Invitrogen) for 2 min at 37 °C, encouraged by gentle trituration by pipette. The suspension was the incubated with 5 μg ml^−1^ DNase I in serum-free L-15 medium for 5 min on ice, before addition of 20 ml L-15/10% FBS-PSG. Single cells were strained through a 40 μm cell strainer, pelleted at 250 g for 5 min, and suspended in fresh L-15/10% FBS-PSG, counted and plated at the desired density.

For flow cytometry and sorting, mammary cell suspensions (10^6^ cells ml^−1^) were stained with anti-CD24-FITC (clone M1/69, BD Biosciences, Oxford, UK; 0.5 μg ml^−1^.), anti-CD45-PE-Cy7 (clone 30-F11, BD Biosciences; 0.25 μg ml^−1^), anti-CD49f-PE-Cy5 (clone GoH3, BD Biosciences; 5.0 μl ml^−1^) and anti-Sca-1-PE (clone D7, BD Biosciences, 0.5 μg ml^−1^) (ref. 34). Cells were sorted at low pressure (20 psi using a 100 μm nozzle) on a FACSAria (Becton Dickenson, UK) equipped with violet (404 nm), blue (488 nm), green (532 nm), yellow (561 nm) and red (635 nm) lasers. Both cell sample and collection tubes were maintained at 4 °C. Single stained samples were used as compensation controls. Dead cells, CD45^+^ leucocytes and non-single cells were excluded. Within the CD24-positive population, two separate cell populations were annotated as CD24^High^ and CD24^Low^ (ref. 53). To standardize gating on CD49f^High^ mammary epithelial cells, we used 5% interval linear density contour plots and considered the outer edge of the main body of the CD24^Low^ cells, as defined by the contour plots, to be the limit above which cells can be considered CD49f^High^ (ref. 33).

### Single-cell mammary organoid culture

Freshly sorted cell populations were plated on NuncTM MicroWellTM 96-Well Optical-Bottom plates in Matrigel (9,500 cells per 9.5 μl) and overlaid with defined basal culture medium, supplemented with Nrg1 (100 ng ml^−1^), Noggin (100 ng ml^−1^) and R-Spondin 1 (2.6 ng ml^−1^; Peprotech). Lower concentrations of recombinant R-spondin 1 from Peprotech were used compared with R-spondin 1 from R&D (100 ng ml^−1^) due to its superior activity. Cultures were additionally treated with Rho kinase inhibitor (Y-27632, 10 μM, Tocris) for the first 5 days. As previously observed for other cell types^13^,^54^, Y-27632 in our hands increased the colony-forming efficiency of single cells.

### Whole-culture immunofluorescence analysis

Whole-culture immunofluorescence was performed following fixing of mammary organoids at stated time points, by application of 4% paraformaldehyde at room temperature for 30 min. Each well was then washed with phosphate-buffered saline (PBS)-Glycine, three times for 10 min, and left overnight at 4 °C in 10% horse serum (Invitrogen) diluted in homemade immunofluorescence buffer (Supplementary Table 4). Primary antibodies, as detailed in Supplementary Table 5, were then applied diluted in immunofluorescence buffer overnight at 4 °C. Following three washes in immunofluorescence buffer, secondary antibodies Alexa Fluor 488 goat anti-mouse IgG (1/500, Invitrogen) and Alexa Fluor 568 goat anti-rabbit IgG (1/500, Invitrogen) were applied overnight at 4 °C. Following three washes in immunofluorescence buffer, Hoechst counterstain was applied for 30 min, and samples images in PBS. Confocal microscopy was performed using a Leica TCS SP2 AOBS spectral confocal microscope, using the 20 × objective. Images were then processed using FIJI software.

### Immunohistochemistry and immunofluorescence

Mammary organoids in matrigel were fixed in 10% formalin, paraffin embedded and cut into 4 μm sections for immunostaining. Briefly, slides were boiled for 20 min in antigen retrieval solution (Dako) followed by blocking in 0.3% hydrogen peroxide for 5 min and goat serum for 45 min (Vector). The antibodies, dilutions and incubation times used in this study are listed in Supplementary Table 5. The specificity of antibodies was validated on normal mammary gland tissue sections (Supplementary Fig. 5). After 1-h incubation with biotinylated secondary antibody, detection was carried out using a Vectastain ABC kit (Vector) for 30 min and visualized with DAB substrate chromogen for 10 min (Sigma).

For immunofluorescence, slides were treated as described above. Slides were then exposed to Alexa Fluor 488 goat anti-mouse IgG (1/1,000, Invitrogen) and Alexa Fluor 568 goat anti-rabbit IgG (1/1,000, Invitrogen) for 1 h and 4′, 6-diamidino-2-phenylindole dihydrochloride (DAPI) stained (1/5,000). Fluorescent images were taken on an IX-71 inverted microscope (Olympus, Essex, UK) via an ORCA-ER camera utilizing SimplePCI software (Hamamatsu Corporation, Hertfordshire, UK). Illumination intensity, exposure, offset and gain settings were maintained between samples. Images were processed using FIJI software.

### Time lapse video-microscopy

Time lapse video-microscopy of mammary organoid growth was carried out using an IX70 inverted DIC microscope (Olympus) at 10 × magnification and recorded using an ORCA-ER CCD camera utilizing SimplePCI v6.0 software (Digitalpixal). Cells were maintained in a temperature-controlled (37 °C), humidified environment in the presence of 5% CO_2_ during imaging.

### β-Galactosidase staining and cell viability

Mammary epithelial organoids were isolated from fourth mammary fat pads of 8–12-week-old virgin Axin2-LacZ mice and cultured under the three different conditions previously described. Wells were washed in PBS, before application of LacZ fixation solution (1:1 mix PBS:buffered formalin (10%) with 4 μl ml^−1^ 25% glutaraldehyde (1% final)) for 1 h at RT. Wells were then washed with PBS twice, and incubated overnight at RT in X-gal staining solution (2.1 mM MgCl_2_, 3 mM K_3_Fe, 3 mM K_4_Fe in PBS, with additional 4 μl per ml 5% (w/v) X-gal in dimethylformamide supplemented). Wells were then washed in PBS and finally fixed in 10% buffered formalin to stop staining, before storage in PBS.

Cell viability was measured using the cell-proliferation reagent WST-1 (Roche). Briefly, mammary organoids in matrigel were incubated with WST-1 reagent for 2 h at 37 °C. The WST-1 solution was then transferred to a 96-well plate and absorbance was measured at 450 nm using a BMG FLUOstar OPTIMA microplate reader.

### Karyotyping

Organoids grown under defined conditions were treated with 0.05 μg ml^−1^ Colcemid (Gibco, Invitrogen) for 16 h to arrest cells in metaphase. Organoids were then washed and trypsinised as standard, before 15 min treatment with a hypotonic solution of potassium chloride (56 mM). Single cells were centrifugated at 1,200 r.p.m. for 4 min and fixed in 3:1 MeOH:Acetic acid by slow addition over vortex. Cells were washed two further times in fixative solution, before plating onto slides to create chromosome spreads. Slides were aged overnight at 55 °C, before staining of chromosomes with DAPI. Images were obtained using an Olympus IX73 40 × microscope, with a 40 × objective. Chromosome spreads were counted manually using the ROI manager plugin of ImageJ software. Compliant with previous publications in which organoid-derived cells have been karyotyped^15^,^16^,^32^, a normal karyotype was defined as 39–41 chromosomes.

### Mutant β-catenin induction in Tet-OΔN89-β-Catenin organoids

Epithelial cells were obtained as described above, from a tetracycline-inducible transgenic mouse line, expressing a constitutively active, truncated β-catenin (ΔN89β-catenin)^42^. Mice were untreated before dissection and experiment start point. Single cells were plated at 1,000 per μl growth-factor reduced Matrigel, and overlaid with media containing either Nrg1 (100 ng ml^−1^) and Noggin (100 ng ml^−1^) alone as a negative control, or supplemented with R-Spondin 1 (2.656 ng ml^−1^), Doxycycline (2 μg ml^−1^) or both in combination. Measurements of organoid number and size were taken at day 7.

### Wnt signalling inhibitor assays

Freshly isolated mammary epithelial cells were seeded in growth-factor reduced Matrigel at 1,000 cells per μl, and overlaid with media containing Nrg1 (100 ng ml^−1^), Noggin (100 ng ml^−1^), R-Spondin 1 (2.656 ng ml^−1^) and a fixed concentration of either IWP-2 (Sigma Aldrich; 0.5, 1, 2 or 4 μM) or IWR-1 (Sigma Aldrich; 37.5, 75, 150 or 300 nM), for 14 days in culture. Organoid number and diameter for each titration was then analysed using GelCountTM software and compared with a DMSO-matched control.

### Lentiviral knockdown of ErbB3/ErbB4

shRNAs were developed using the Block-It shRNA system (Thermo Fisher) against both Erbb3 and Erbb4 cloned into a Gateway modified pSEW vector (Groner Lab). Lentiviral pseudo-virus was produced using the psPAX2 and PMD.G2 lentiviral packaging plasmids (Trono lab). Lentiviral titre was established and test cells were infected at an MOI of >3.

For infection, freshly isolated single mammary epithelial cells were resuspended in an equal mix of Matrigel and lentiviral suspension, and plated over a pre-set thin layer of 100% Matrigel in 48-well plates. Each well was then overlaid with culture media containing Nrg1 (100 ng ml^−1^), Noggin (100 ng ml^−1^) and R-Spondin 1 (2.656 ng ml^−1^). After 24 h to allow cells to settle into the Matrigel and infection to occur, media was replaced. This process was repeated every 2 days.

Plates were fixed after 10 days and counterstained with DAPI, and images taken with Olympus IX73 microscope, with a 10 × objective. Organoid number and size was analysed using ImageJ.

### HGF addition assays

Freshly isolated mammary epithelial cells were seeded in growth-factor reduced Matrigel at 1,000 cells per μl, and overlaid with media containing Nrg1 (100 ng ml^−1^), Noggin (100 ng ml^−1^), R-Spondin 1 (2.656 ng ml^−1^) and a fixed concentration of HGF (Peprotech; 3.125, 6.25, 12.5, 25 or 50 ng ml^−1^). At 11 days in culture, organoid number and diameter per well was then analysed using GelCountTM software and compared with an untreated control.

### Cleared mammary fat pad transplantation

Mammary organoids were cultured for 30 days under the 3 different culture conditions described. In all, 50 organoids were transplanted per mammary fat pad of 3-week-old Rag-1 or FvB mice from which the endogenous epithelium was surgically removed^53^. Eight weeks after transplantation, fat pads were whole-mounted, stained with carmine and analysed. Failed clears were excluded from the analysis. Wholemounts were examined on a binocular microscope (Zeiss, Stemi SV11). Images were captured with a Canon PC1089 camera and Canon Zoom Browser software (with white balance set to fluorescent and auto exposure activated).

### RNA isolation and qPCR

Freshly sorted normal cell populations were resuspended in RLT buffer. Total RNA was isolated using RNeasy Mini kit (Qiagen) following the manufacturer's recommendations. DNA was eliminated using gDNA spin columns during RNA isolation and gDNA Wipeout Buffer during cDNA retrotranscription. cDNA synthesis was performed using QuantiTect Reverse Transcription Kit (Qiagen). Quantitative real-time PCR was carried out using the following TaqMan assays: Mm00695835_m1 (Erbb3), Mm01256814_m1 (Erbb4) and Mm00626552_m1 (Nrg1). Gene assays were done on triplicates. Three experimental replicates were done using different animal sets.

For *in vitro* experiments, mammary organoids cultured for 30 days were treated with oestrogen (4 ng ml^−1^; Sigma Aldrich), progesterone (40 ng ml^−1^; Sigma Aldrich) or both for 4 or 24 h in phenol red free conditions. Matrigel was mechanically dissociated and organoids were pelleted by centrifugation at 1,000 r.p.m. for 3 min. RNA was extracted using the RNeasy micro kit (Qiagen). In all, 1 μg of total RNA was reverse transcribed using random hexamers (Promega) and Superscript III reverse transcriptase (Invitrogen). The qRT-PCR was performed using FastSybr Green Master Mix kit (Applied Biosystems). Duplicate samples were analysed on a StepOnePlus (Applied Biosystems) qRT-PCR machine using the fast protocol. β-2 microglobulin, ribosomal protein L13A (RPL13A) and hypoxanthine phosphoribosyltransferase were selected as normalizers. Threshold cycle (CT) values and the 2-ΔΔCT method were used to calculate expression changes. Supplementary Table 6 contains the list of primers used in this study.

### Data availability

The data supporting the findings of this study are available within the article and its Supplementary Information Files. All other relevant source data are available from the authors on request.

## Additional information

**How to cite this article:** Jardé, T. *et al*. Wnt and Neuregulin1/ErbB signalling extends 3D culture of hormone responsive mammary organoids. *Nat. Commun.* **7,** 13207 doi: 10.1038/ncomms13207 (2016).

## Supplementary Material

Supplementary Information Supplementary Figures 1-27 and Supplementary Tables 1-6.

Supplementary Movie 1 Time-lapse microscopy of a mammary organoid grown in Neuregulin 1 conditions. Mammary epithelial cells were embedded in growth-factor reduced matrigel and overlaid with basal culture medium supplemented with Noggin (100 ng/ml) and Neuregulin 1 (100 ng/ml). After 16 days in culture, images were captured every hour, for approximately 96hrs. Frame rate: 15 frames per second.

Supplementary Movie 2 Time-lapse microscopy of a mammary organoid grown in Neuregulin 1 / R-spondin 1 conditions. Mammary epithelial cells were embedded in growth-factor reduced matrigel and overlaid with basal culture medium supplemented with Noggin (100 ng/ml), R-spondin 1 (20 ng/ml) and Neuregulin 1 (100 ng/ml). After 16 days in culture, images were captured every hour, for approximately 96hrs. Frame rate: 15 frames per second.
