# Supplementary material for: Wnt and Neuregulin1/ErbB signalling extends 3D culture of hormone responsive mammary organoids
Source: Nat Commun. 2016 Oct 26;7:13207. doi: 10.1038/ncomms13207 (PMC5095178; doi:10.1038/ncomms13207)
Supplement: Supplementary Information — Supplementary Figures 1-27 and Supplementary Tables 1-6. [file ncomms13207-s1.pdf]

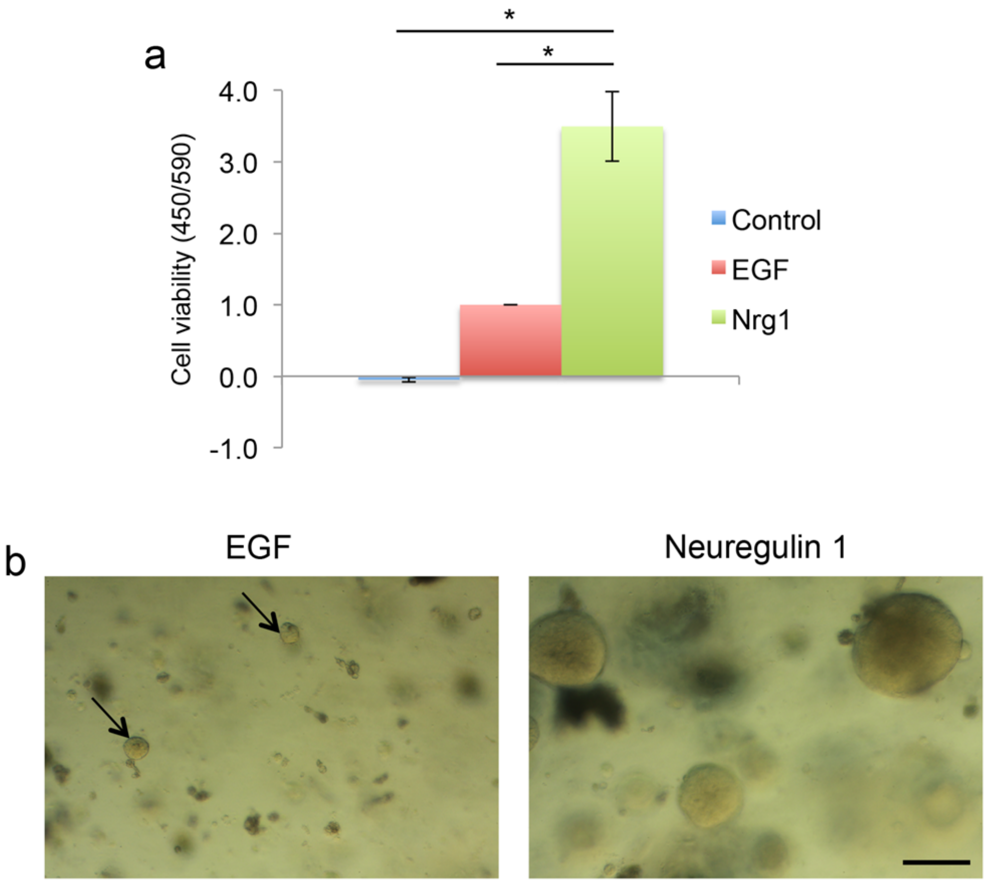

Supplementary Figure 1: Neuregulin 1 increases the growth of mammary organoids compared to EGF. (a) Mammary epithelial cells were freshly isolated, embedded in matrigel and exposed to culture medium containing Noggin (100ng/ml), EGF (100 ng/ml) or Neuregulin 1 (100ng/ml) for 15 days. The number of viable cells (Wst assay) was evaluated (n=3, means±s.e.m.). *, $p < 0.05$, paired Student T test. (b) Representative pictures of mammary organoids treated with culture medium containing Noggin (100ng/ml), EGF (100 ng/ml) or Neuregulin 1 (100ng/ml) for 15 days. Scale bar, 50 µm.

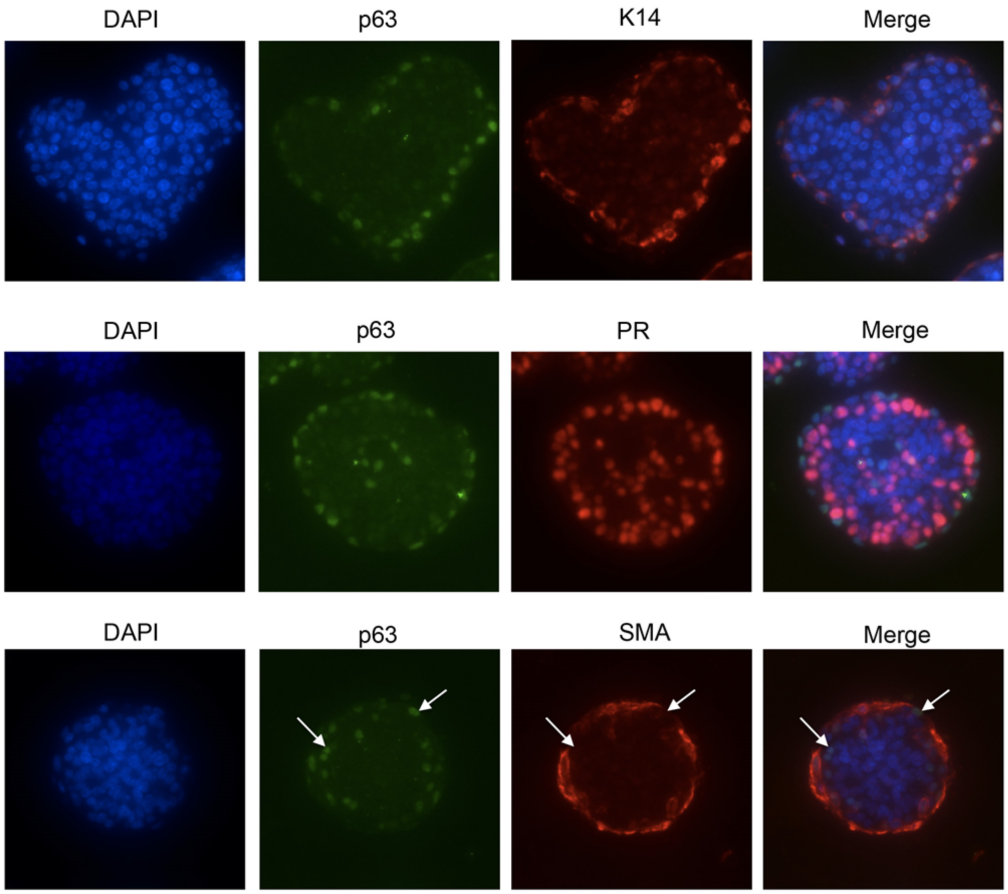

Supplementary Figure 2: Neuregulin 1 treated organoids contain distinct cell compartments. Mammary epithelial cells were cultured with 100 ng/ml Neuregulin 1 and 100 ng/ml Noggin for 30 days. Organoids were fixed, embedded in paraffin and sectioned. Organoid sections were stained for p63, keratin 14 (K14), progesterone receptor (PR) and smooth muscle actin (SMA). Note the presence of p63+ SMA- cells (white arrows) in the basal cell layer. Counterstain, DAPI (blue).

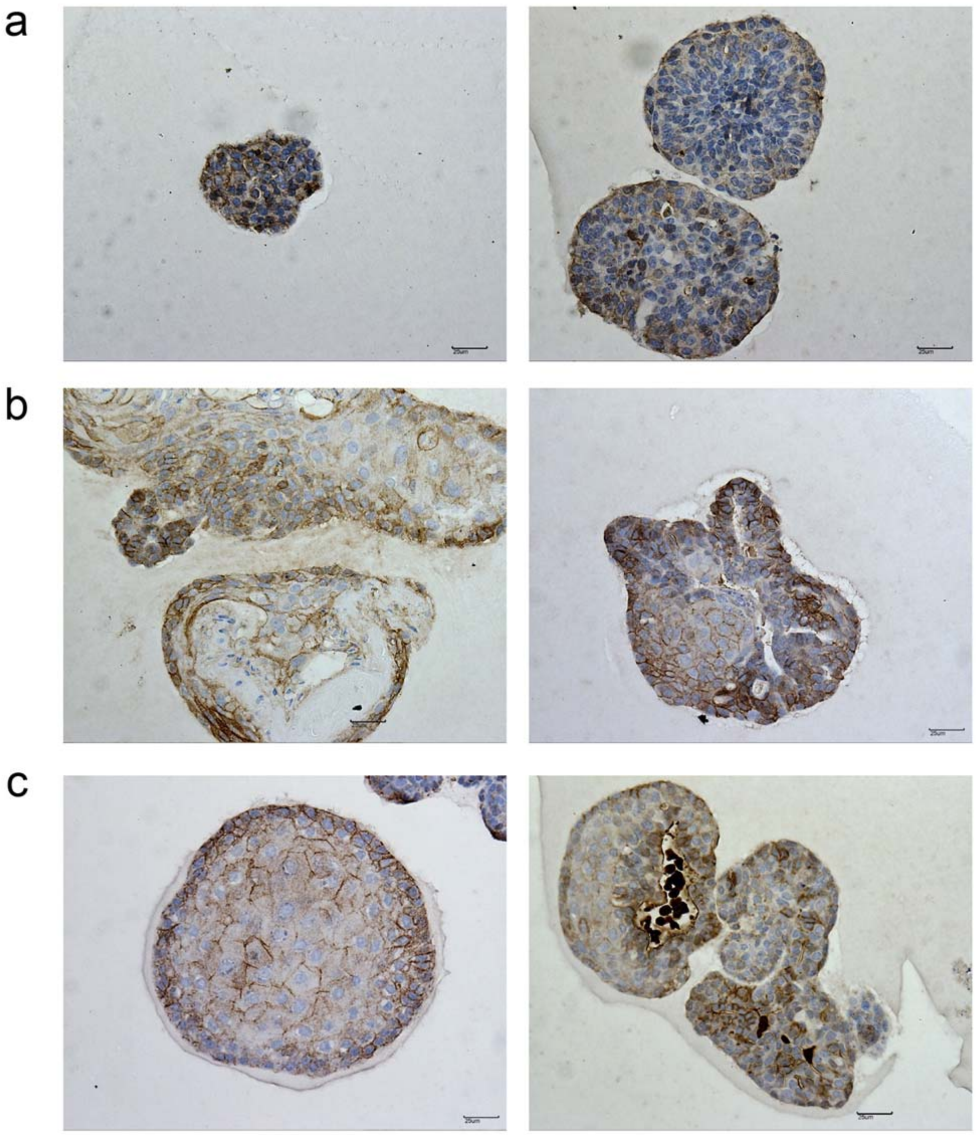

Supplementary Figure 3: Immunohistochemical detection of β-catenin in mammary organoids grown under Neuregulin 1 (100ng/ml) / Noggin (100ng/ml) (a), EGF (50ng/ml) / Noggin (100ng/ml) / R-spondin 1 (600ng/ml) (b) and Neuregulin 1 (100ng/ml) / Noggin (100ng/ml) / R-spondin 1 (100ng/ml) (c) culture conditions. Scale bar, 25μm.

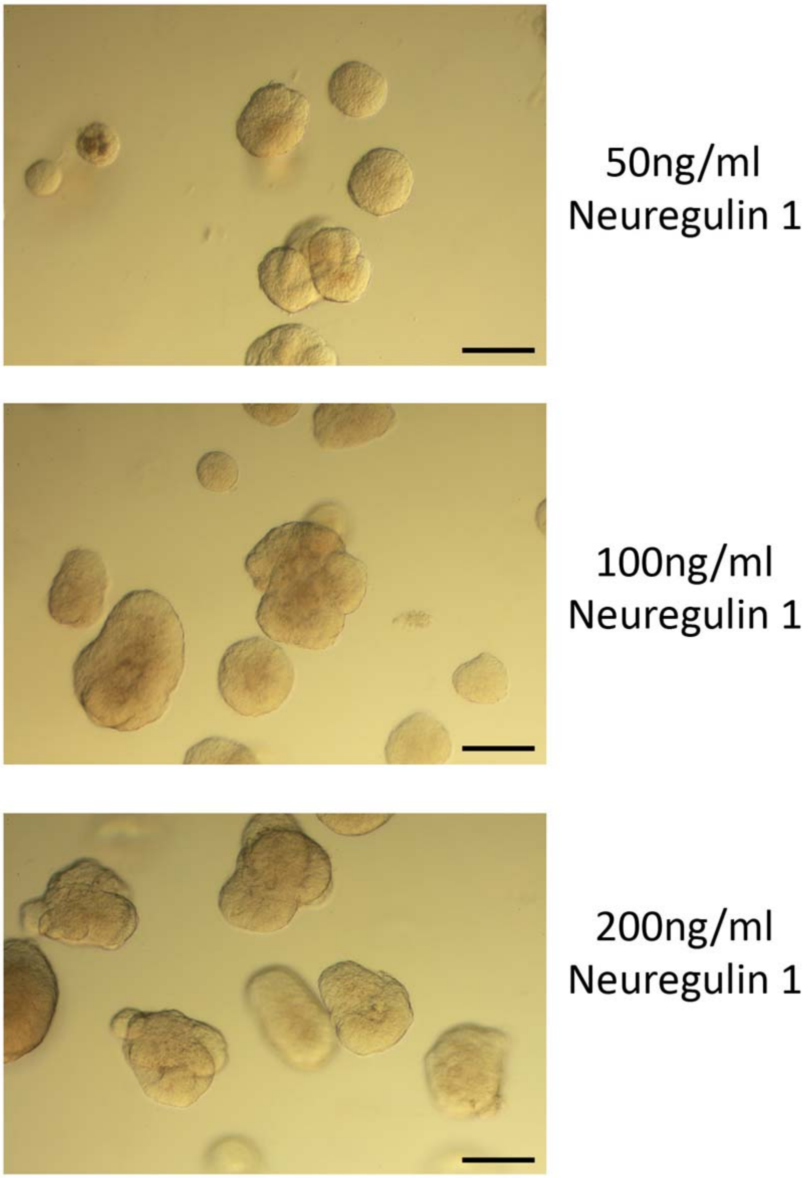

Supplementary Figure 4: High concentrations of Neuregulin 1 promotes the growth of mammary organoids compared to low concentrations of Neuregulin 1. Mammary epithelial cells were freshly isolated, embedded in matrigel and exposed to culture medium containing Noggin (100ng/ml) and increasing concentrations of Neuregulin 1 for 30 days. Scale bar, 100 µm.

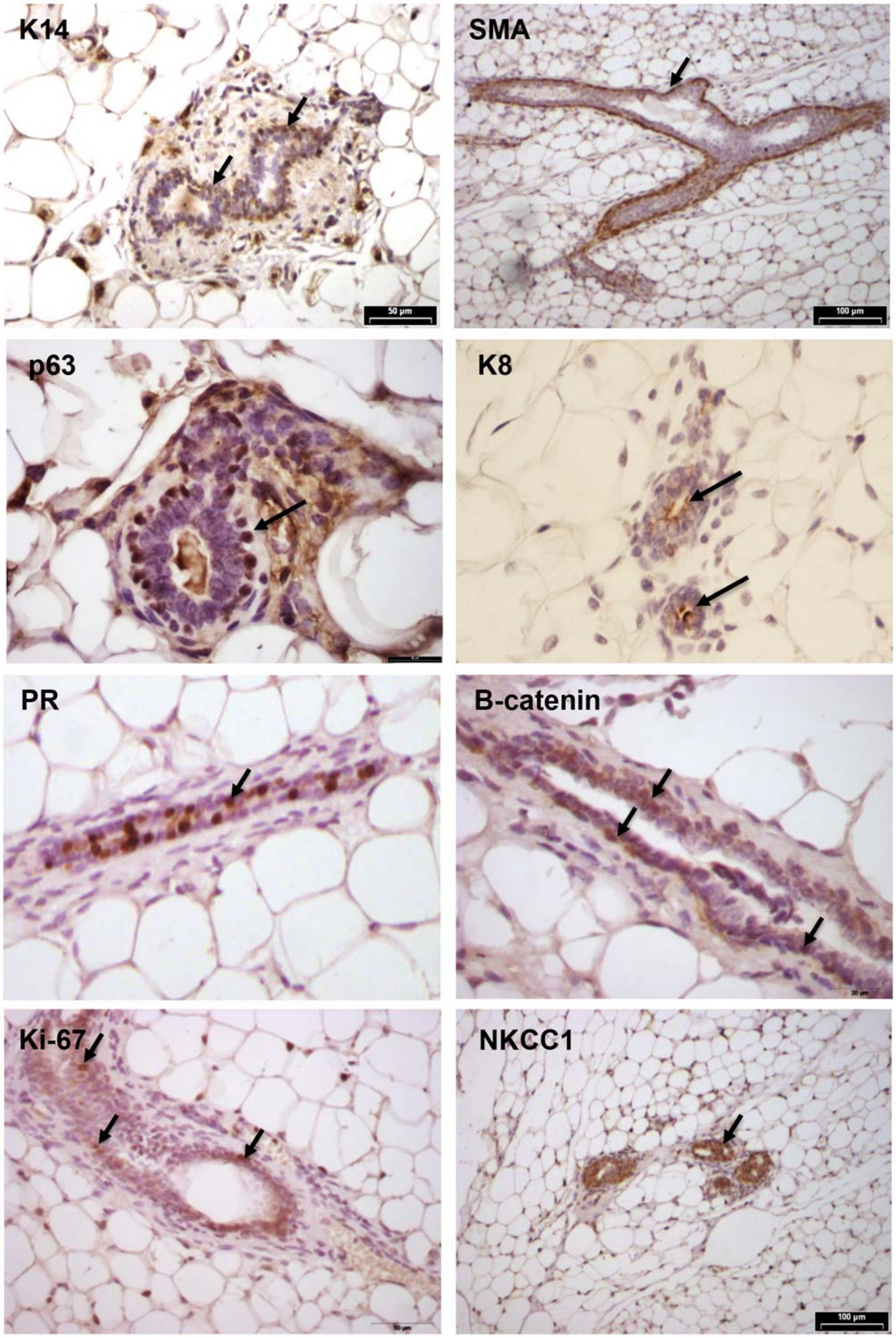

Supplementary Figure 5: Validation of antibodies used in this study on mouse mammary gland tissue.

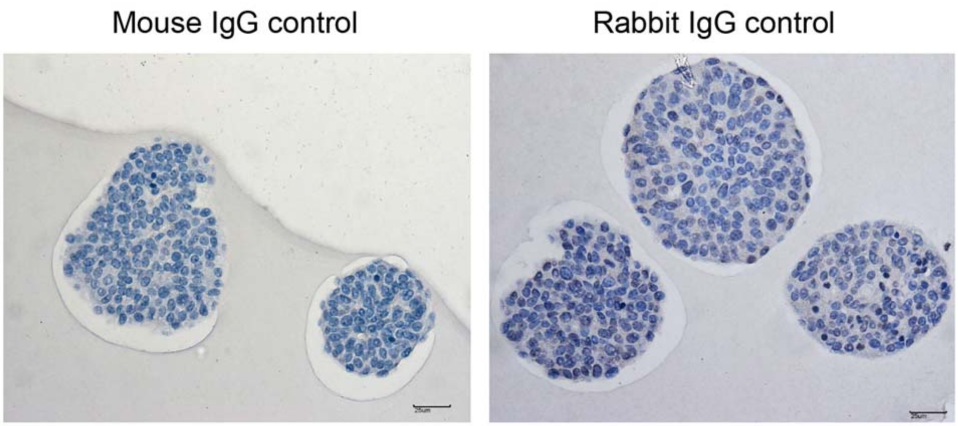

Supplementary Figure 6: Mammary organoid sections exposed to mouse IgG or rabbit IgG control antibodies show no immunohistochemical staining. Scale bar, 25 μm.

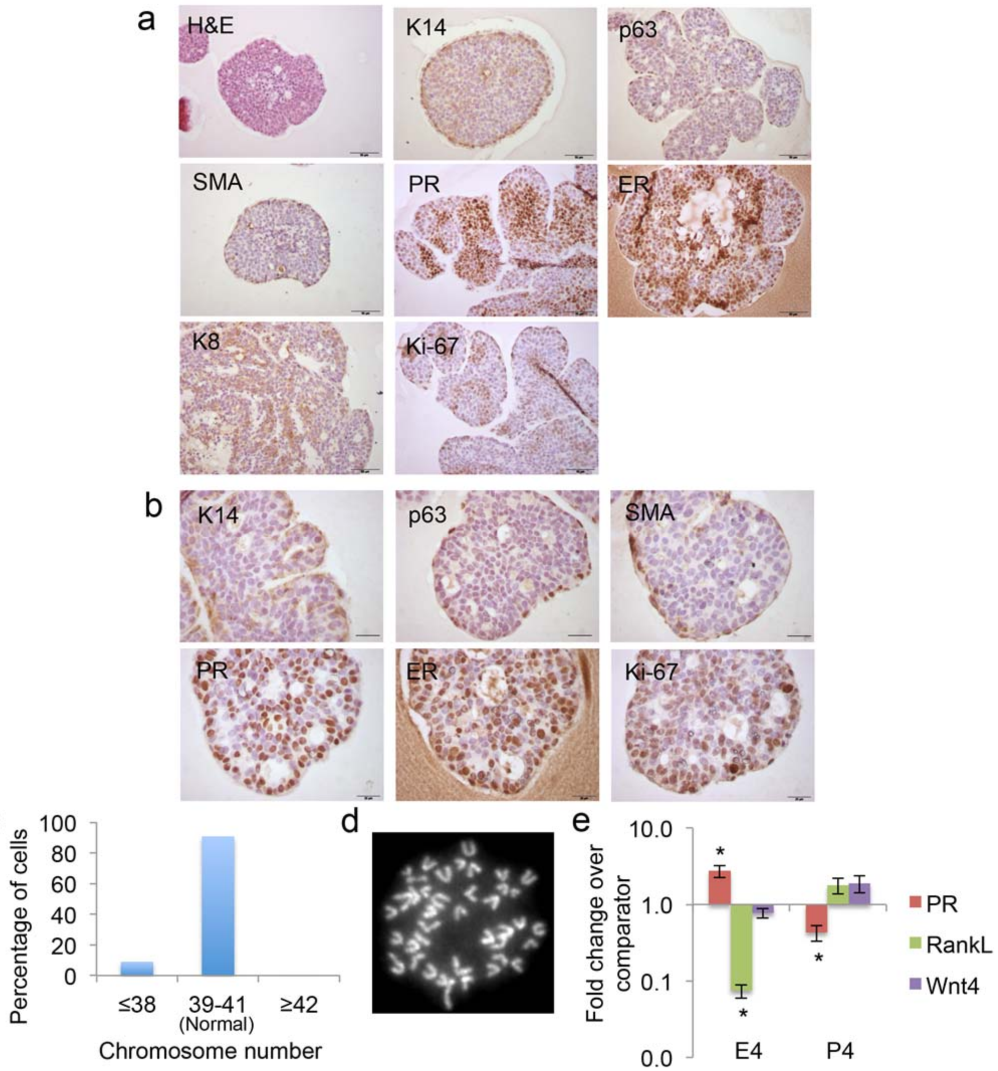

Supplementary Figure 7: Mammary organoids treated with Neuregulin 1 can be maintained in culture for long-term. Mammary epithelial cells were freshly isolated, embedded in matrigel, exposed to culture medium containing Neuregulin 1 (100ng/ml) and Noggin (100ng/ml) and cultured for 75 days. Mammary organoids were then fixed and stained for selected markers. (a) Scale bar, 50 µm. (b) Scale bar, 20 µm. (c) Quantification of chromosome number in mammary organoids cultured for 75 days (n=11). Chromosomal spreads with 40±1 chromosomes were considered normal. (d) Representative picture of metaphase chromosome spread that shows a normal number of chromosomes. (e) Expression of PR, RankL and Wnt4 following treatment with estrogen (E, 4ng/ml) or progesterone (P, 40ng/ml) for 4 hours. The gene expression was evaluated by quantitative RT-PCR. Data are expressed as fold change (vs untreated, n=3, means±s.e.m.). *, $p < 0.05$, paired Student T test.

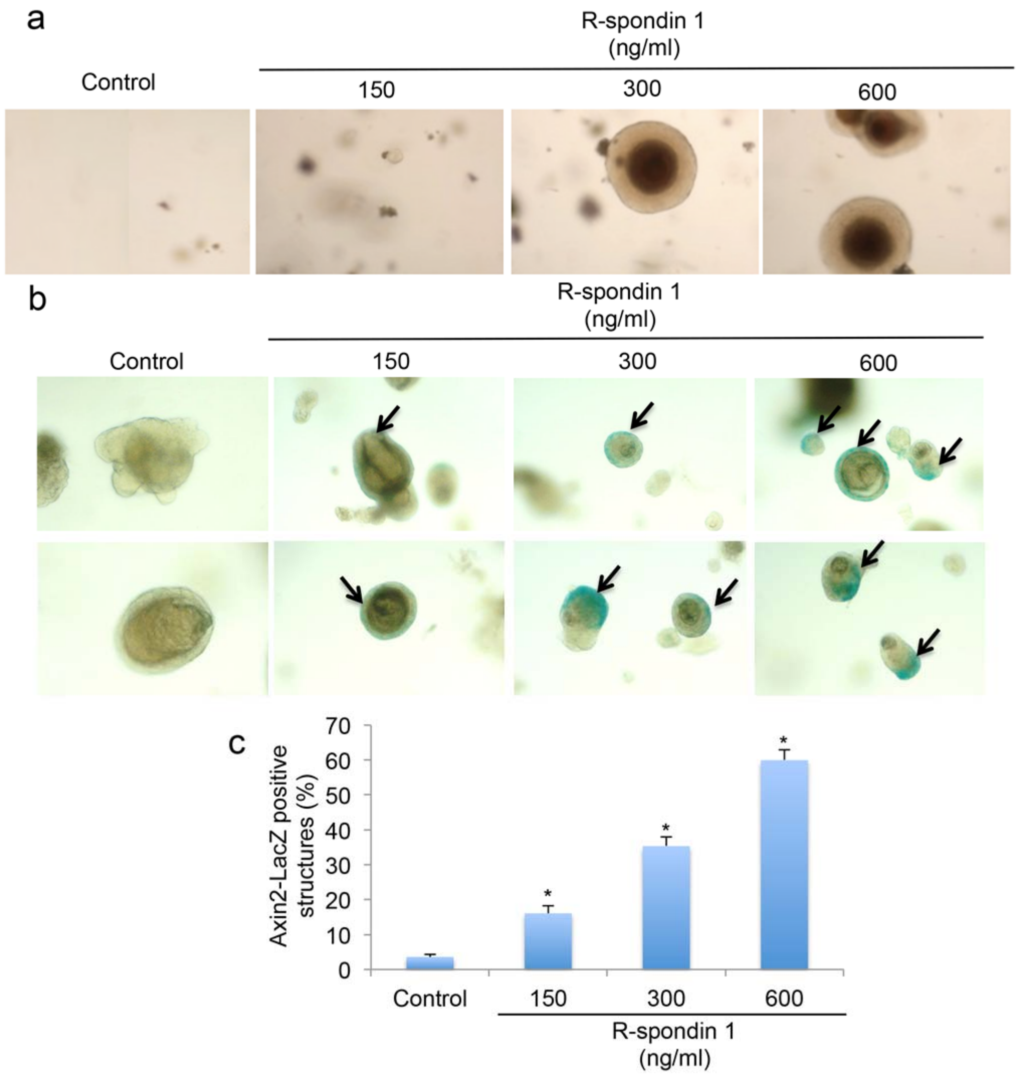

Supplementary Figure 8: R-spondin 1 regulates mammary organoid formation and Wnt signalling activation. (a) High concentrations of R-spondin 1 (300 and 600 ng/ml) promote the formation of complex round mammary structures. (b) Axin2-LacZ mammary epithelial cells were cultured for 5 months with 600 ng/ml R-spondin 1 (the culture medium contained also 50 ng/ml EGF and 100 ng/ml Noggin). The medium was replaced with a R-spondin 1 free culture medium for 3 days. Mammary organoids were then treated with increasing concentrations of R-spondin 1 for 2 days, fixed and stained for β-galactosidase. (c) Quantification of axin2-LacZ positive mammary organoids (n=3, means±s.e.m.). *, $p<0.05$, paired Student T test.

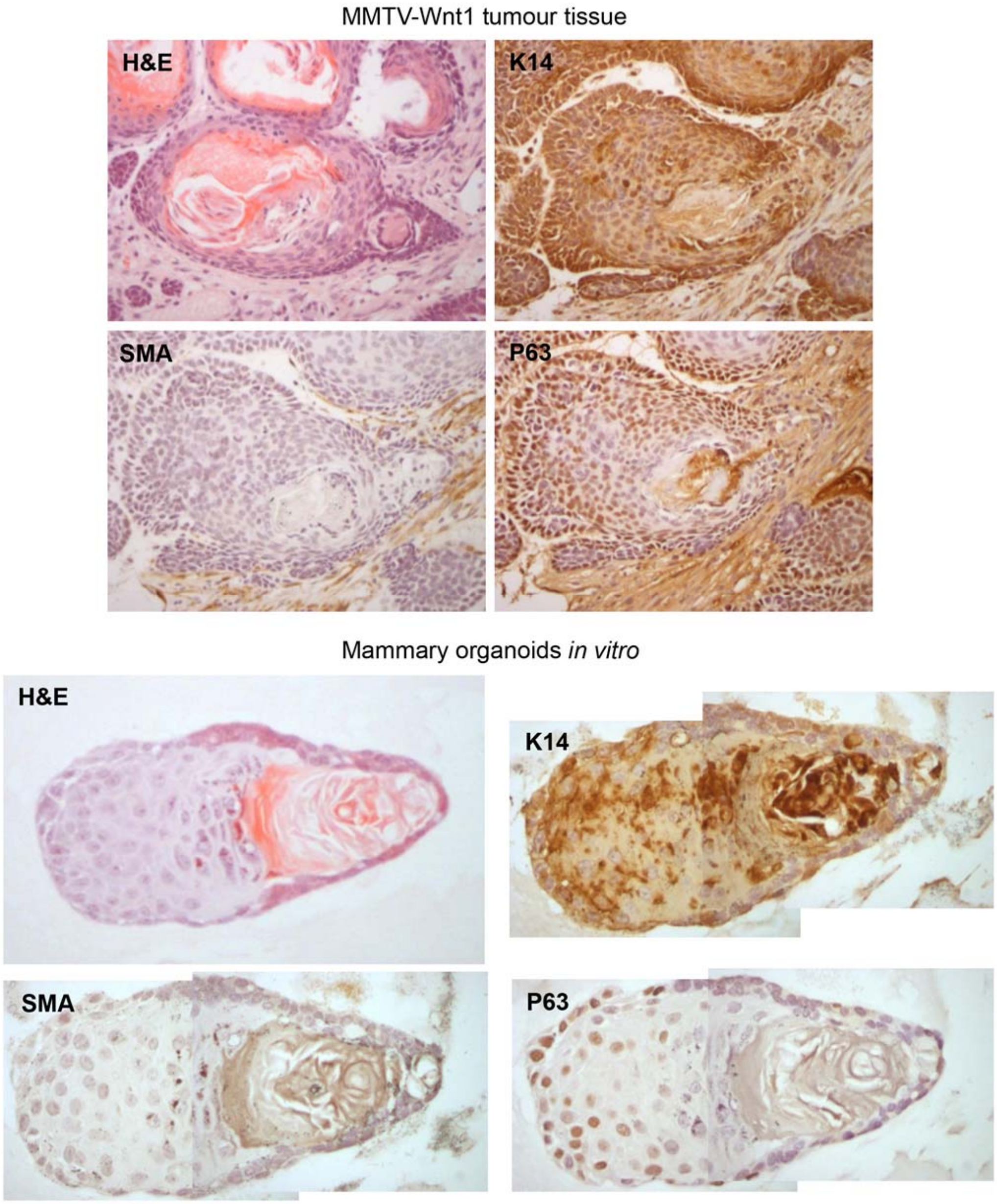

Supplementary Figure 9: R-spondin 1 treated mammary organoids recapitulate MMTV-Wnt1 tumour tissue histology. Mammary epithelial cells were cultured with 600 ng/ml R-spondin 1, 50 ng/ml EGF and 100 ng/ml Noggin for 21 days. Organoids were fixed, embedded in paraffin and sectioned. Sections were stained with Hematoxylin and Eosin (H&E) and for basal markers (keratin-14, K14; p63; smooth muscle actin, SMA). In parallel, MMTV-Wnt1 driven mammary gland tumours were fixed, embedded in paraffin, sectioned and stained.

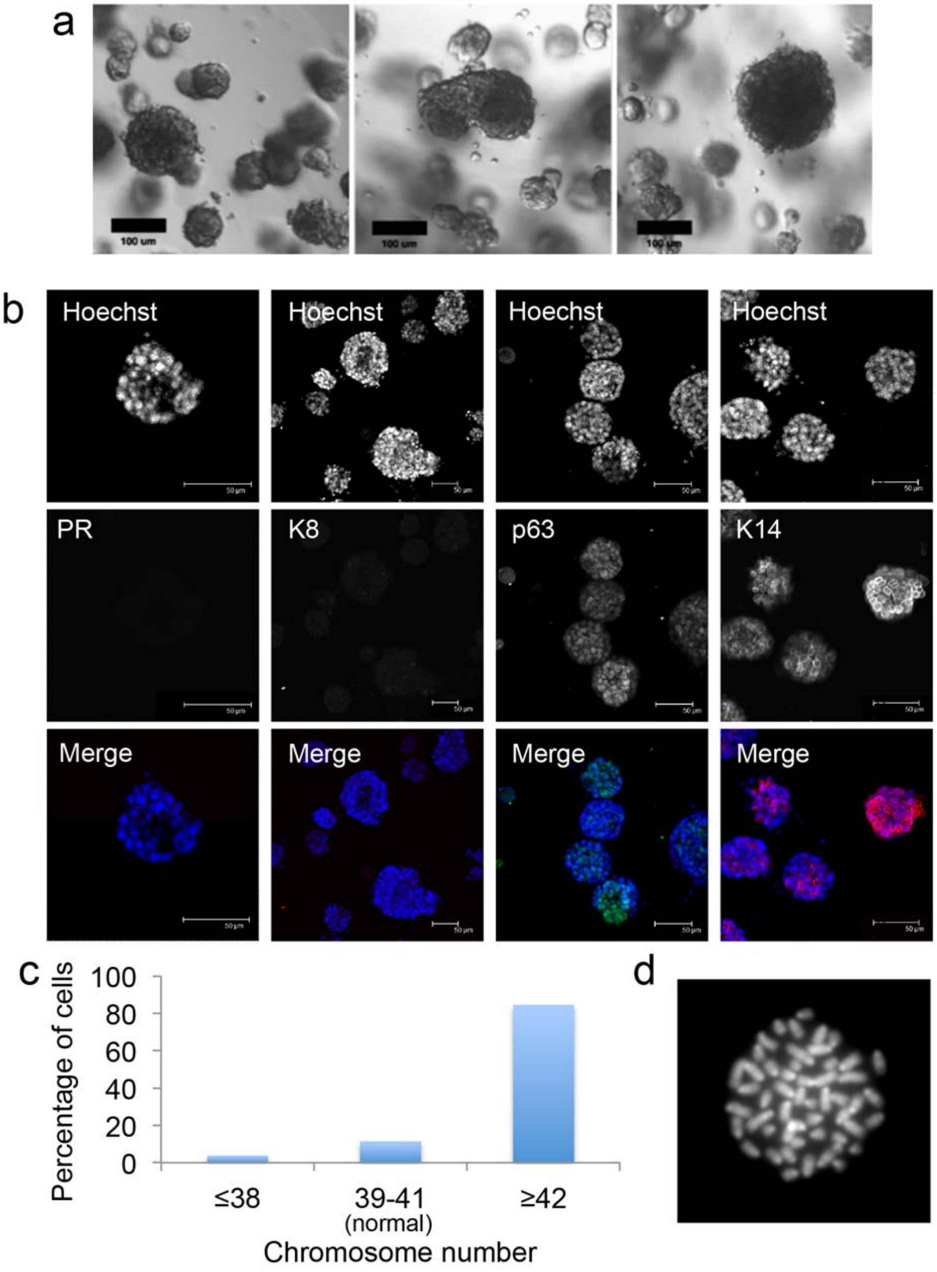

Supplementary Figure 10: Long-term culture of mammary organoids with EGF and high concentrations of R-spondin 1 is associated with chromosomal abnormalities. Mammary epithelial cells were freshly isolated, embedded in matrigel, exposed to culture medium containing EGF(50ng/ml), Noggin (100ng/ml) and R-spondin 1 (42.5 ng/ml) and cultured for 100 days (passaged 10 times). (a) Representative pictures of mammary organoids (scale bar, 100 µm). (b) Mammary organoids were fixed and stained for the basal markers p63 and keratin 14 (K14) and luminal markers progesterone receptor (PR) and keratin 8 (K8) (DAPI, blue) (scale bar, 50 µm). (c) Quantification of chromosome number in mammary organoids cultured for 100 days (n=26). Chromosomal spreads with 40±1 chromosomes were considered normal. (d) Representative picture of metaphase chromosome spread that shows an aberrant number of chromosomes.

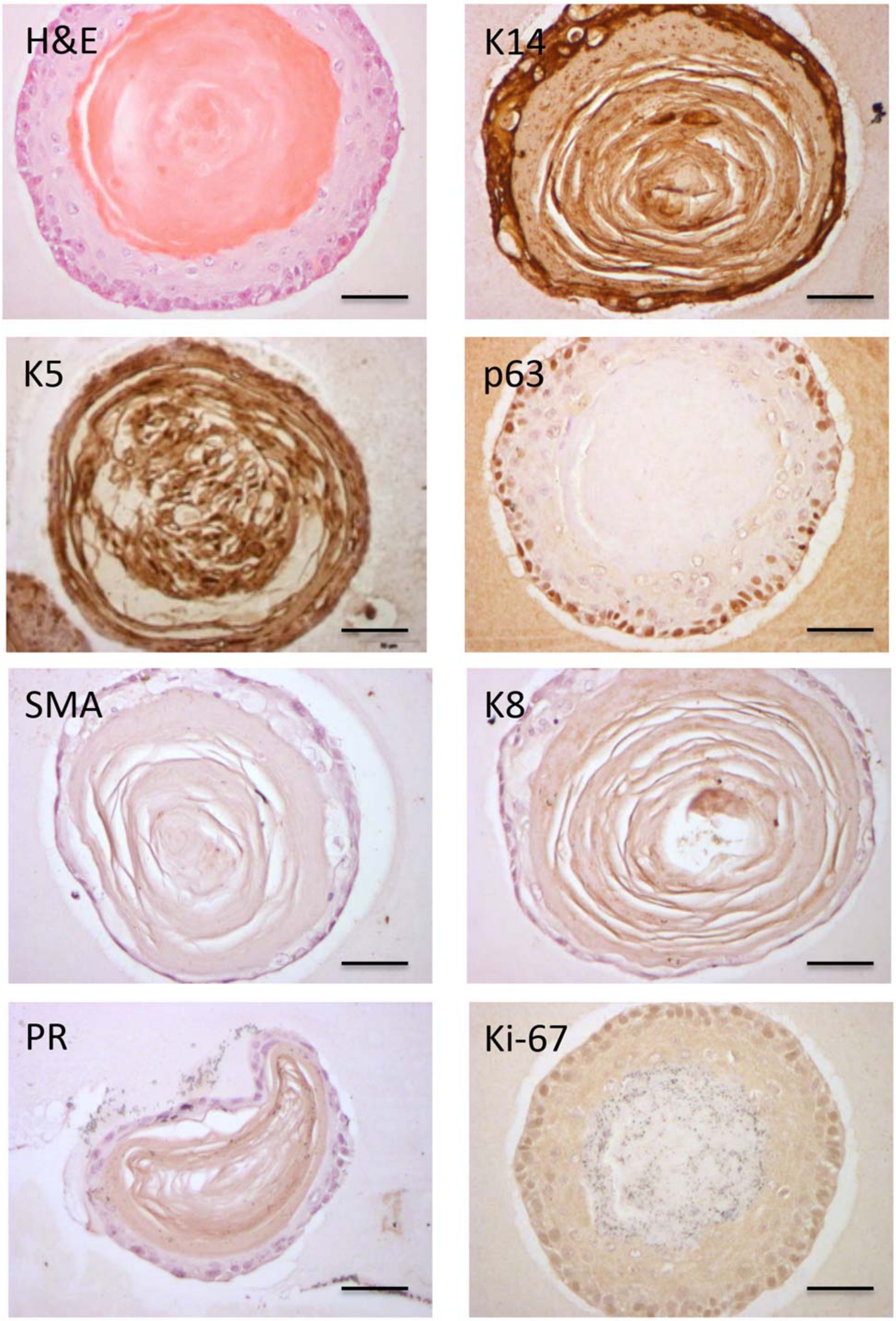

Supplementary Figure 11: Mammary organoids treated with EGF and high concentrations of R-spondin 1 can be maintained in culture for long-term. Mammary epithelial cells were freshly isolated, embedded in matrigel, exposed to culture medium containing EGF(50ng/ml), Noggin (100ng/ml) and R-spondin 1 (600 ng/ml) and cultured for 5 months. Mammary organoids were then fixed and stained for selected markers (scale bar, 50 μm).

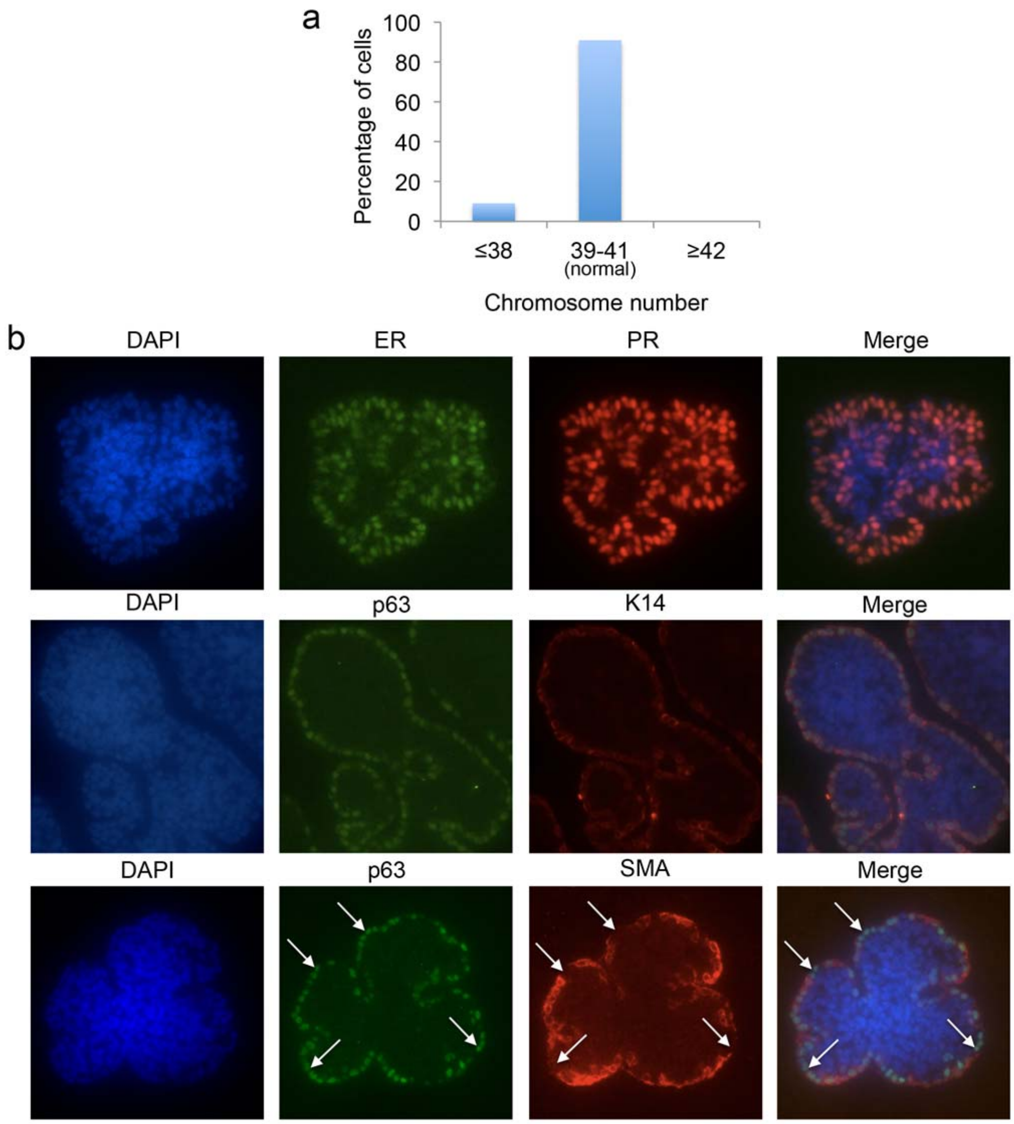

Supplementary Figure 12: R-spondin 1 and Neuregulin 1 support the growth of karyotypically normal organoids that contain a robust basal cell population. Mammary epithelial cells were cultured with 100 ng/ml R-spondin 1, 100 ng/ml Neuregulin 1 and 100 ng/ml Noggin for 30 days. (a) Quantification of chromosome number in mammary organoids cultured for 30 days (n=11). Chromosomal spreads with 40±1 chromosomes were considered normal. (b) Organoids were fixed, embedded in paraffin and sectioned. Organoid sections were co-stained for estrogen receptor (ER), progesterone receptor (PR), p63, keratin 14 (K14) and smooth muscle actin (SMA). Note the presence of p63+ SMA- cells (white arrows) in the basal cell layer. Counterstain, DAPI (blue).

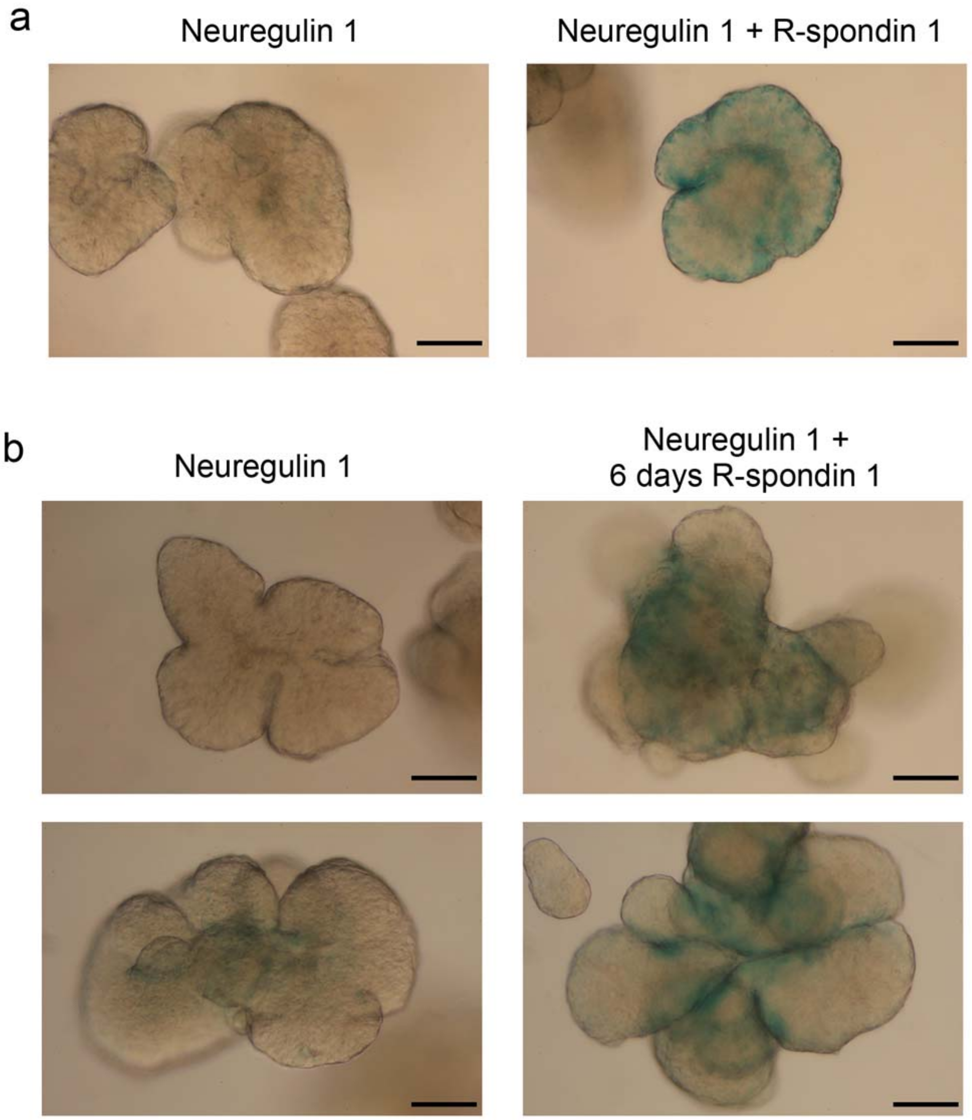

Supplementary Figure 13: R-spondin 1 supports Wnt signalling activation in mammary organoids. (a) Axin2-LacZ (Wnt reporter) mammary epithelial cells were freshly isolated, embedded in matrigel and exposed to culture medium containing Neuregulin 1 (100ng/ml), Noggin (100ng/ml) +/- R-spondin 1 (100 ng/ml) for 30 days. Mammary organoids were then fixed and stained for β-galactosidase. (b) Axin2-LacZ (Wnt reporter) mammary epithelial cells were freshly isolated, embedded in matrigel and exposed to culture medium containing Neuregulin 1 (100ng/ml) and Noggin (100ng/ml) for 6 weeks. The culture medium was then supplementated with or without R-spondin 1 (100 ng/ml) for 6 days. Mammary organoids were fixed and stained for β-galactosidase. Scale bar, 50 μm.

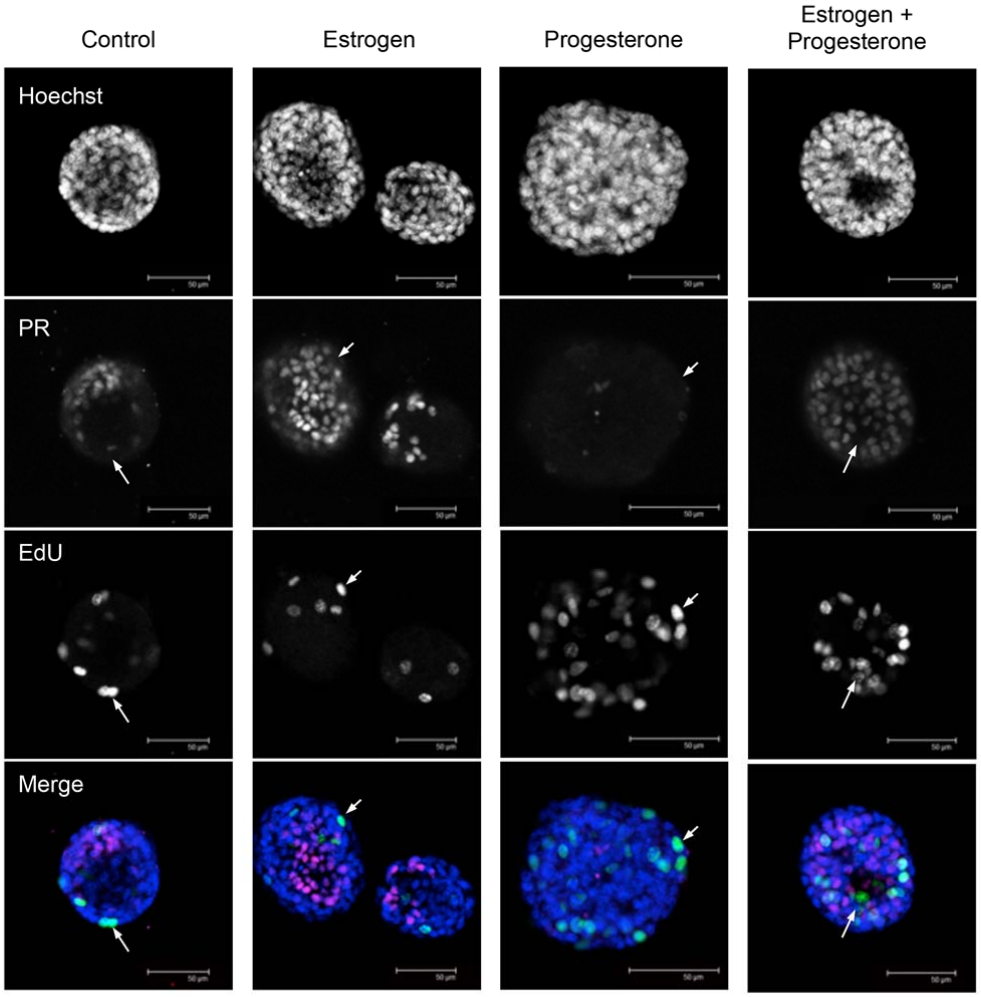

Supplementary Figure 14: Characterisation and identification of proliferating cells within mammary organoids under standard conditions, or following 24 hour treatment with steroid hormones. Mammary organoids were cultured with 2.6 ng/ml R-spondin 1 (Peprotech), 100 ng/ml Neuregulin 1 and 100 ng/ml Noggin for 14 days. Where indicated, estrogen (4ng/ml), progesterone (40 ng/ml) or both hormones were added for the final 24 hours in culture. 10 µM EdU was applied 2 hours prior to fixing, and fixed organoids stained for EdU and progesterone receptor (PR). Counterstain, Hoechst (blue). Scale bars, 50 µm. White arrows indicate distinct examples of EdU positive, PR negative cells.

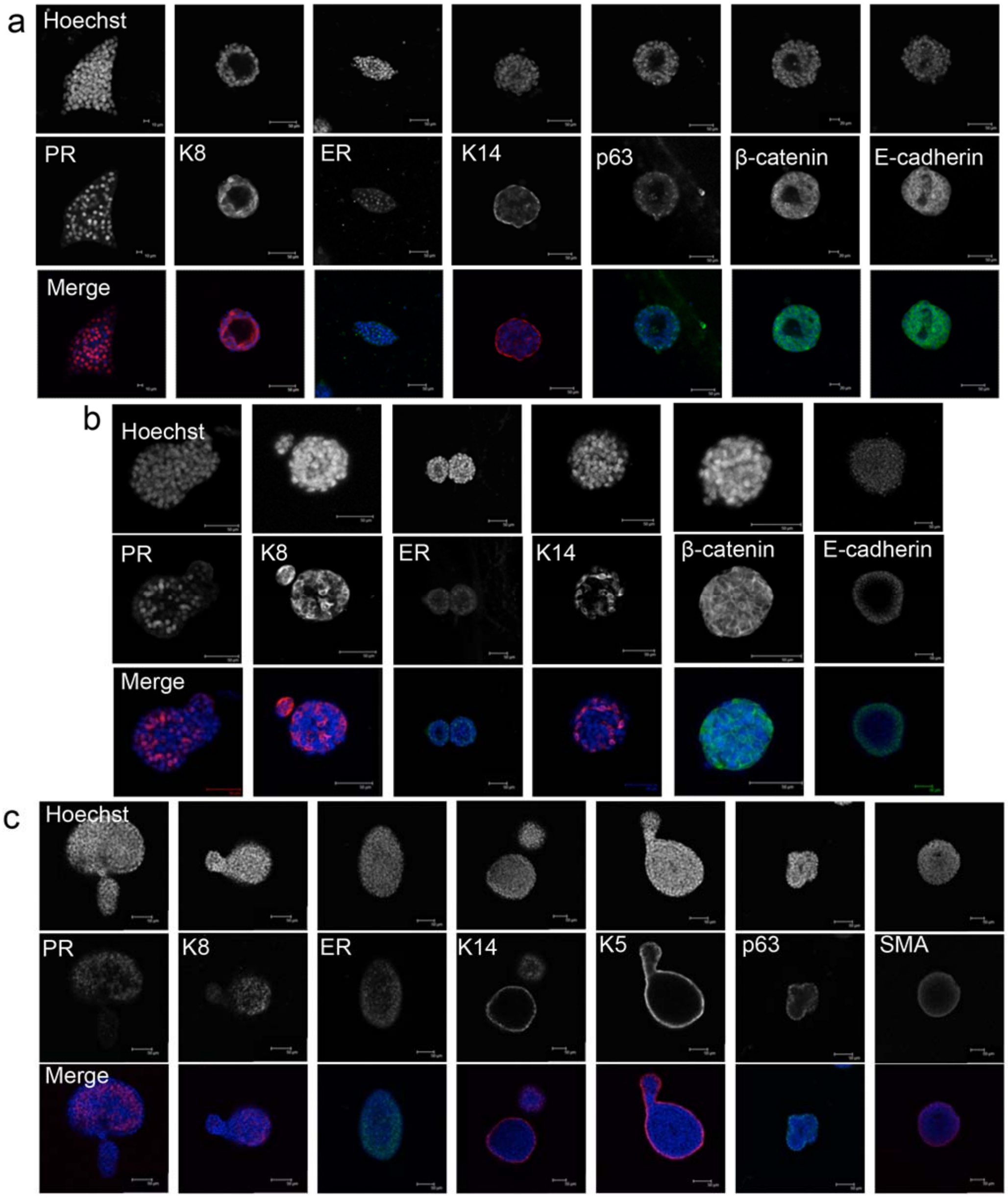

Supplementary Figure 15: Histological characterisation of mammary organoids during development in culture. Mammary organoids were cultured with 2.6 ng/ml R-spondin 1 (Peprotech), 100 ng/ml Neuregulin 1 and 100 ng/ml Noggin for 3.5 days (a), 7 days (b) or 14 days (c). Organoids were fixed and stained for progesterone receptor (PR), keratin 8 (K8), estrogen receptor (ER), keratin 14 (K14), keratin 5 (K5), p63, smooth muscle actin (SMA), β-catenin and E-cadherin. Counterstain, hoechst (blue). Scale bars, 50 µm.

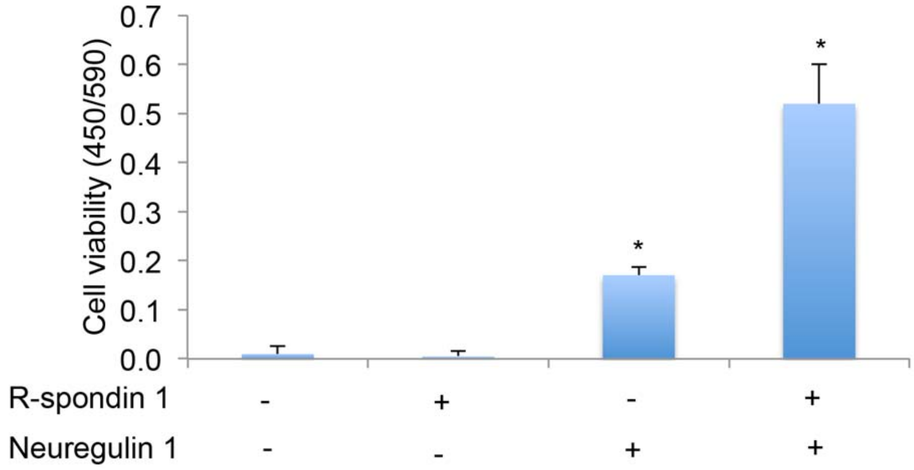

Supplementary Figure 16: R-spondin 1 alone does not promote the growth of mammary organoids. Mammary epithelial cells were cultured with R-spondin 1 (100 ng/ml), Neuregulin 1 (100 ng/ml) or both for 30 days. The number of viable cells (Wst assay) was then measured (n=3, means±s.e.m.). *, $p<0.05$, paired Student T test.

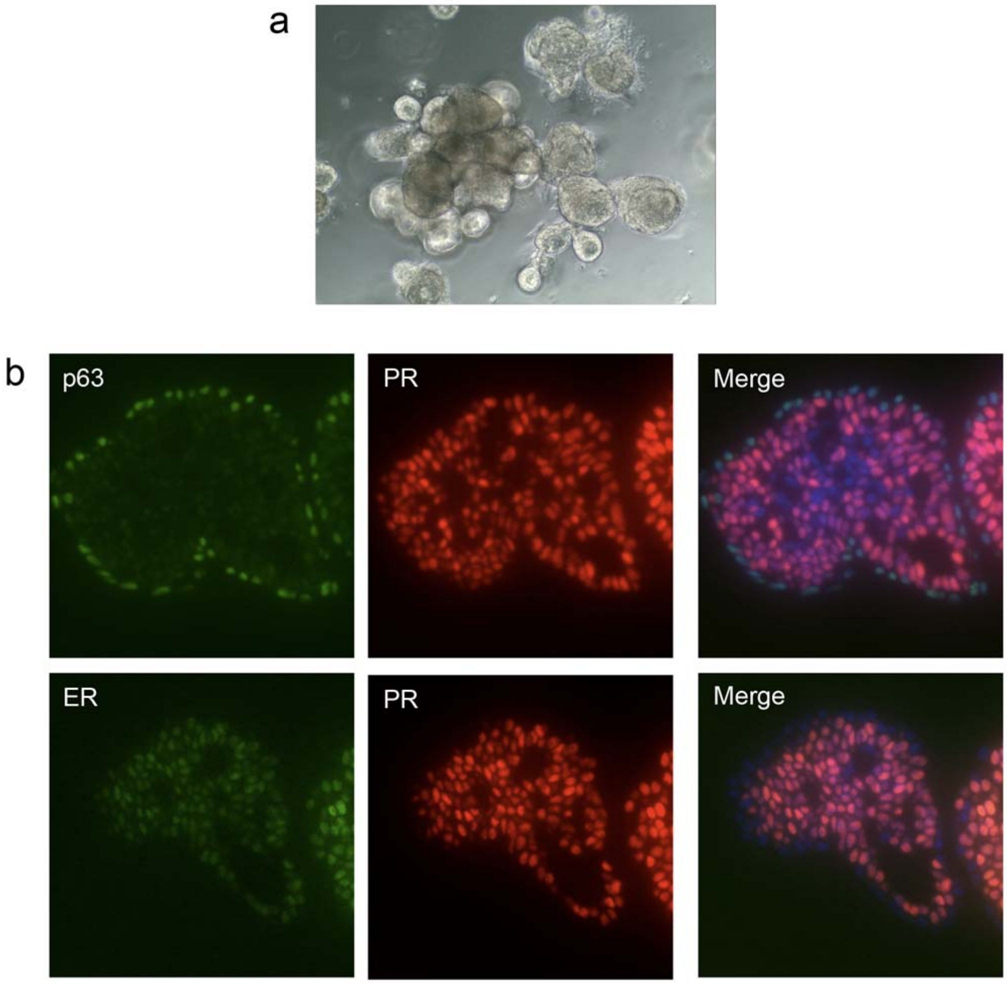

Supplementary Figure 17: Mammary cells from C57BL/6 mouse strain can form organoids with distinct luminal and basal cell compartments. Mammary epithelial cells were freshly isolated, embedded in matrigel and exposed to culture medium containing Neuregulin 1 (100ng/ml), Noggin (100ng/ml) and R-spondin 1 (100 ng/ml). (a) Representative pictures of mammary organoids cultured for 21 days. (b) After 30 days in culture, C57BL/6 mammary organoids were fixed and stained for the basal marker p63 and luminal markers progesterone receptor (PR) and estrogen receptor (ER) (DAPI, blue).

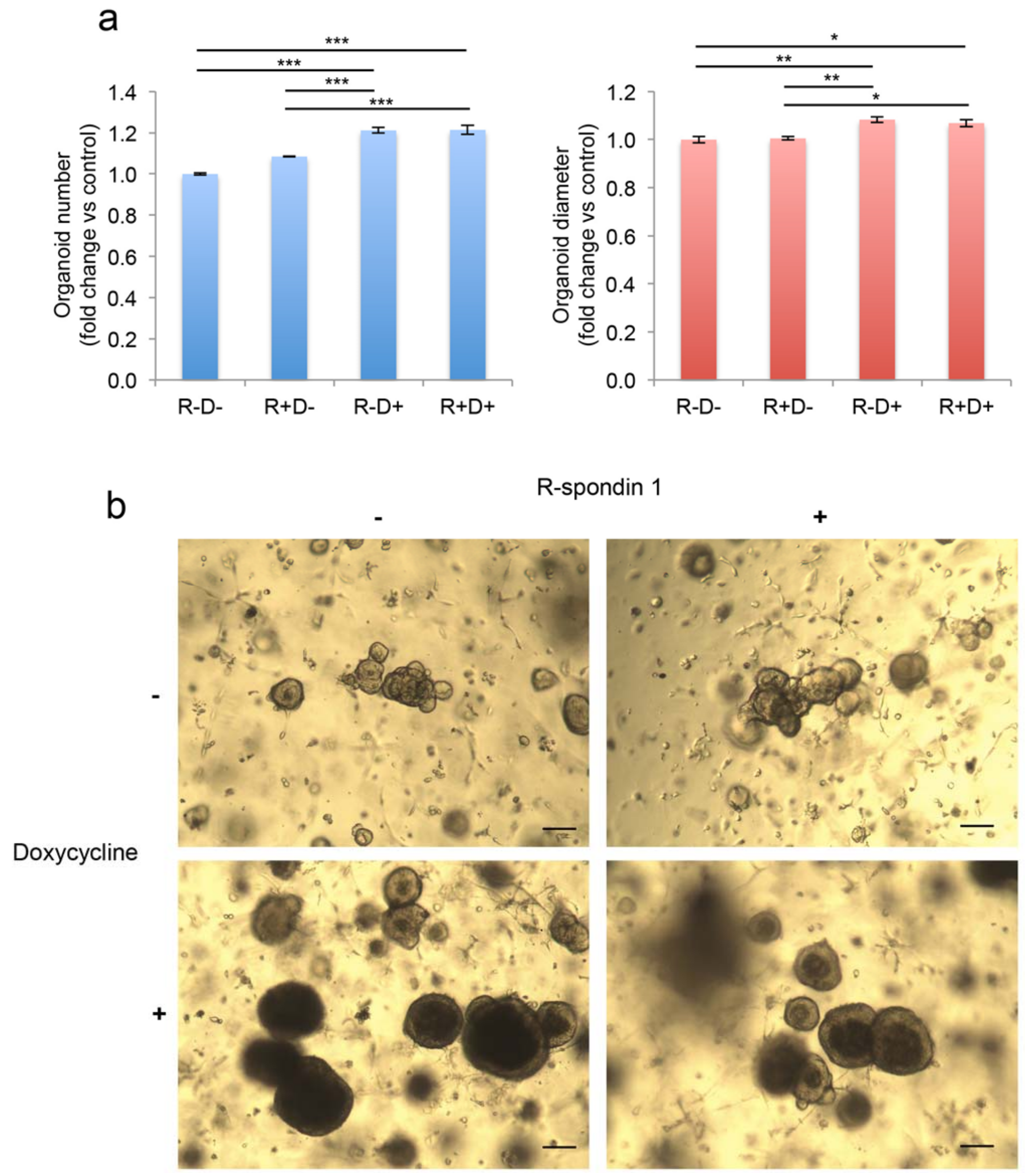

Supplementary Figure 18: Constitutive Wnt pathway activation increases organoid formation and size. Mammary epithelial cells were freshly isolated from a Tet-O-ΔN89β-Catenin mouse line and embedded in matrigel. Cultures were treated with Neuregulin 1 (100 ng/ml) and Noggin (100 ng/ml) supplemented media alone, or additionally given R-Spondin 1 (R; 2.656 ng/ml), Doxycyline (D; 2 µg/ml), or both in combination. (a) After 7 days, organoid number and average diameter were assessed using GelCount™ scanner and software from Oxford Optronix. Data are expressed as fold change (vs Neu, Nog only, n=3, means±s.e.m.). **, $p < 0.01$; ***, $p < 0.001$, ANOVA with Tukey's post-hoc comparison. (b) Representative pictures of mammary organoids cultured for 7 days under each condition. Scale bar, 100 µm.

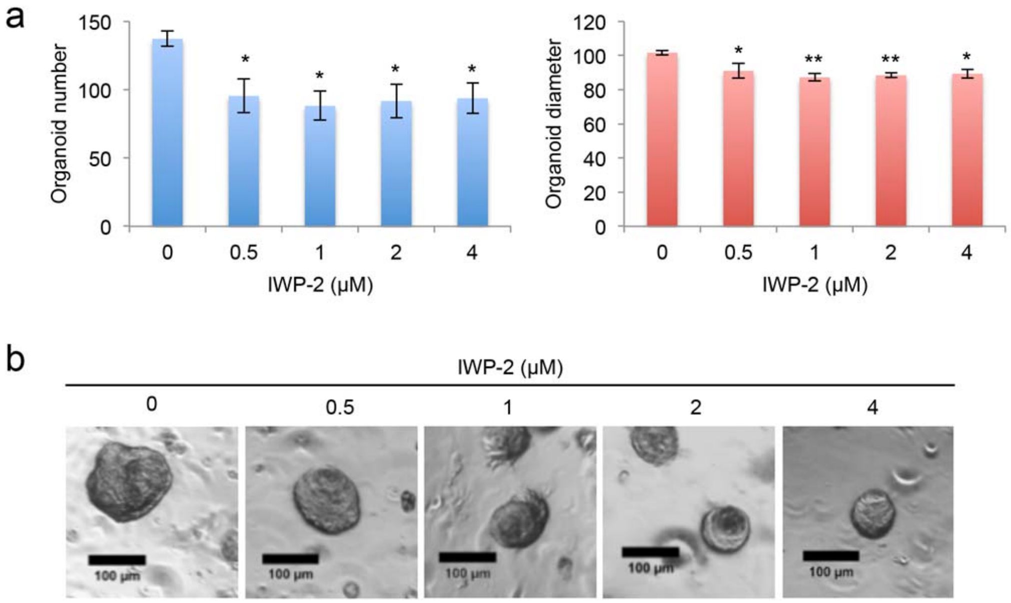

Supplementary Figure 19: The Wnt signalling inhibitor IWP-2 decreases organoid forming efficiency and organoid diameter. Mammary epithelial cells were freshly isolated, embedded in matrigel and exposed to culture medium containing Neuregulin 1 (100ng/ml), Noggin (100ng/ml), R-spondin 1 (2.656 ng/ml) and increasing concentrations of IWP-2. (a) After 14 days, the number and diameter of organoids was assessed using GelCount™ scanner and software from Oxford Optronix. Data are expressed as fold change (vs untreated, n=3, means±s.e.m.). **, $p < 0.01$; ***, $p < 0.001$, ANOVA with Dunnett's post-hoc comparison. (b) Representative pictures of mammary organoids cultured 14 days in the presence of increasing concentrations of IWP-2. Scale bar, 100 µm.

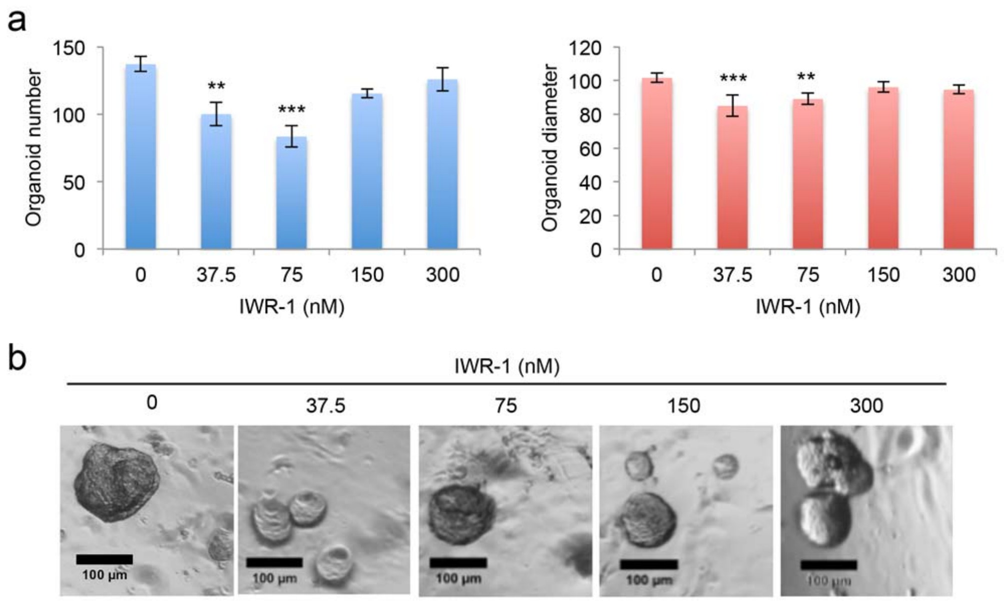

Supplementary Figure 20 The Wnt signalling inhibitor IWR-1 decreases organoid forming efficiency and organoid diameter. Mammary epithelial cells were freshly isolated, embedded in matrigel and exposed to culture medium containing Neuregulin 1 (100ng/ml), Noggin (100ng/ml), R-spondin 1 (2.656ng/ml) and increasing concentrations of IWR-1. (a) After 14 days, the number and diameter of organoids was assessed using GelCount^{TM} scanner and software from Oxford Optronix. Data are expressed as fold change (vs untreated, n=3, means±s.e.m.). **, $p < 0.01$; ***, $p < 0.001$, ANOVA with Dunnett's post-hoc comparison. (b) Representative pictures of mammary organoids cultured 14 days in the presence of increasing concentrations of IWR-1. Scale bar, 100 µm.

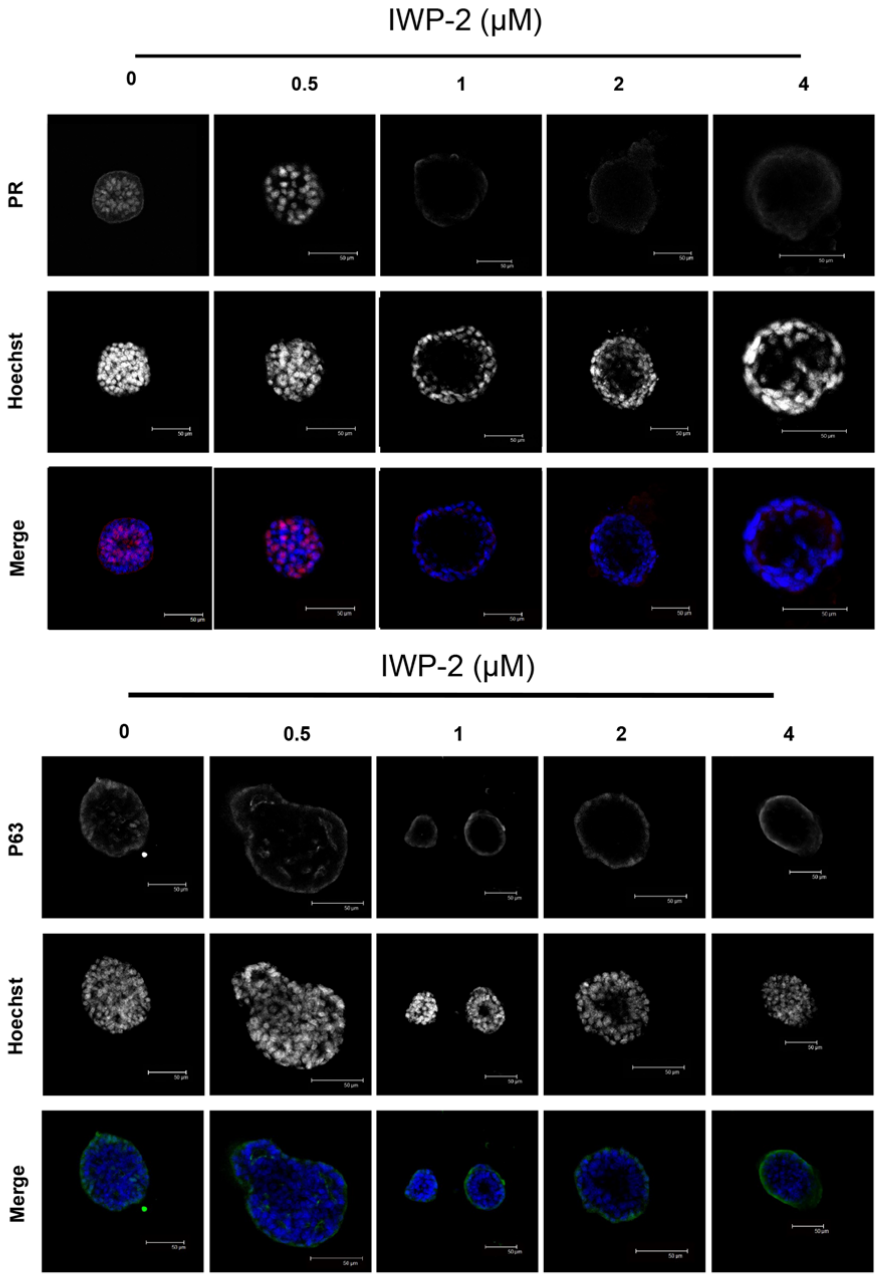

Supplementary Figure 21: The Wnt signalling inhibitor IWP-2 induces loss of expression of PR in mammary organoids. Mammary epithelial cells were freshly isolated, embedded in matrigel and exposed to culture medium containing Neuregulin 1 (100ng/ml), Noggin (100ng/ml), R-spondin 1 (2.6565ng/ml) and increasing concentrations of IWP-2. After 14 days in culture, mammary organoids were fixed and stained for the luminal marker PR and basal marker p63 (counterstain, hoechst). Scale bar, 50 µm.

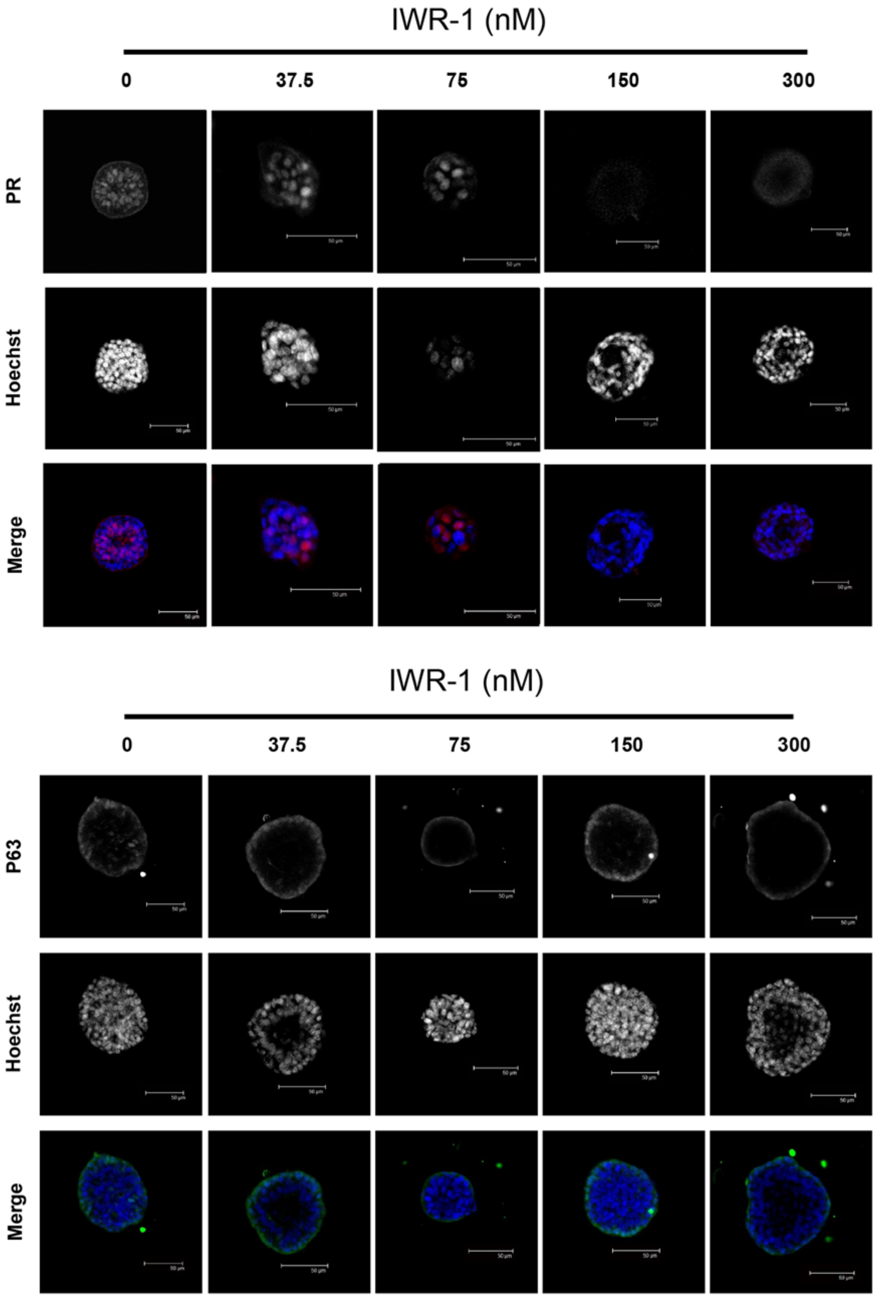

Supplementary Figure 22: The Wnt signalling inhibitor IWR-1 induces loss of expression of PR in mammary organoids. Mammary epithelial cells were freshly isolated, embedded in matrigel and exposed to culture medium containing Neuregulin 1 (100ng/ml), Noggin (100ng/ml), R-spondin 1 (2.656 ng/ml) and increasing concentrations of IWR-1. After 14 days in culture, mammary organoids were fixed and stained for the luminal marker PR and basal marker p63 (counterstain, hoechst). Scale bar, 50 µm.

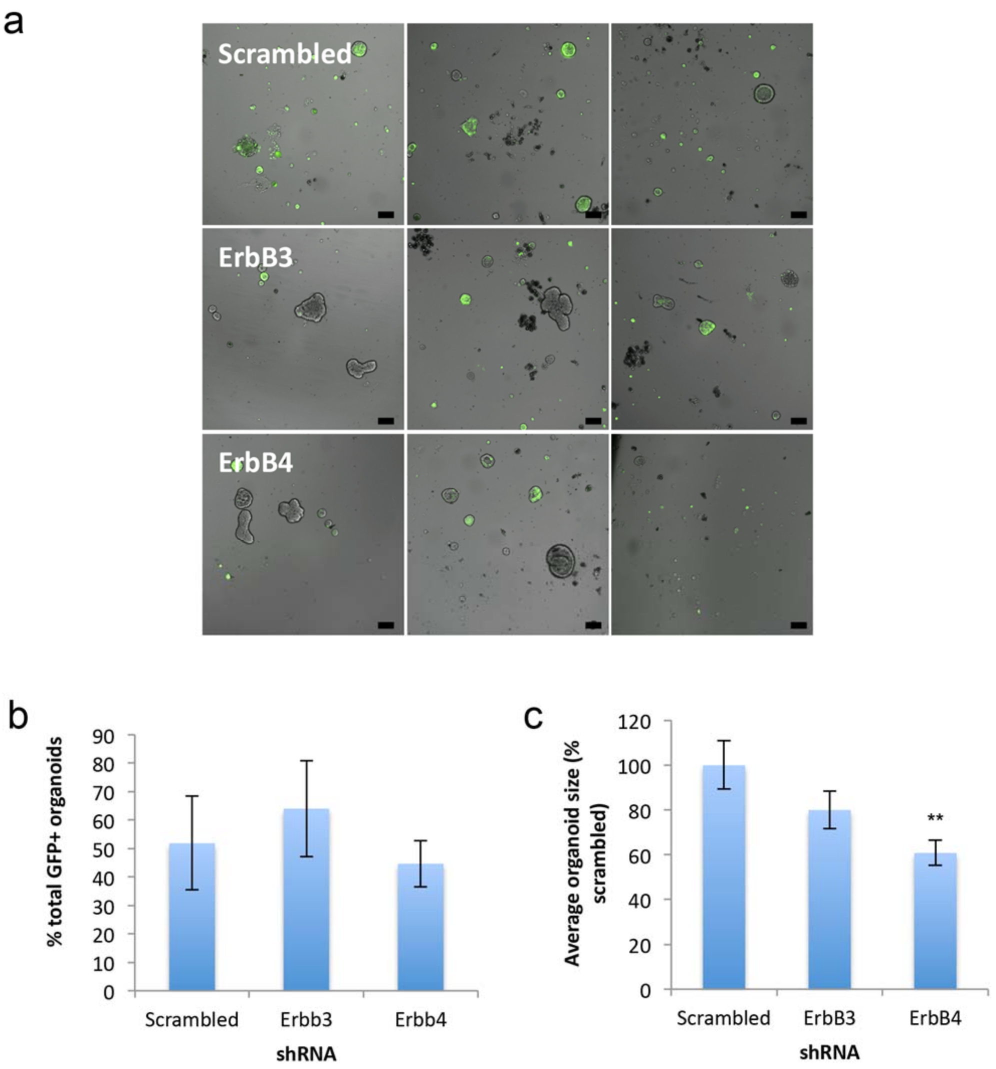

Supplementary Figure 23: Loss of ErbB3 or ErbB4 abrogates mammary organoid growth. Mammary epithelial cells were freshly isolated and infected with control shRNA (scrambled) or ErbB3 or ErbB4 shRNA lentiviruses. Organoids were fixed at day 10 in culture. (a) Representative images of organoids infected with lentivirus, where successful infection was indicated by GFP positivity. Scale bar, 100µm. (b) Organoid infection success was calculated as number GFP+ organoids/total number organoids. Data shown as mean ± (n=3). (c) Average GFP+ organoid area was analysed using ImageJ. Data are expressed as % of scrambled control (mean ± S.E.M, n=3). **, $p<0.01$, Kruskal-Wallis test.

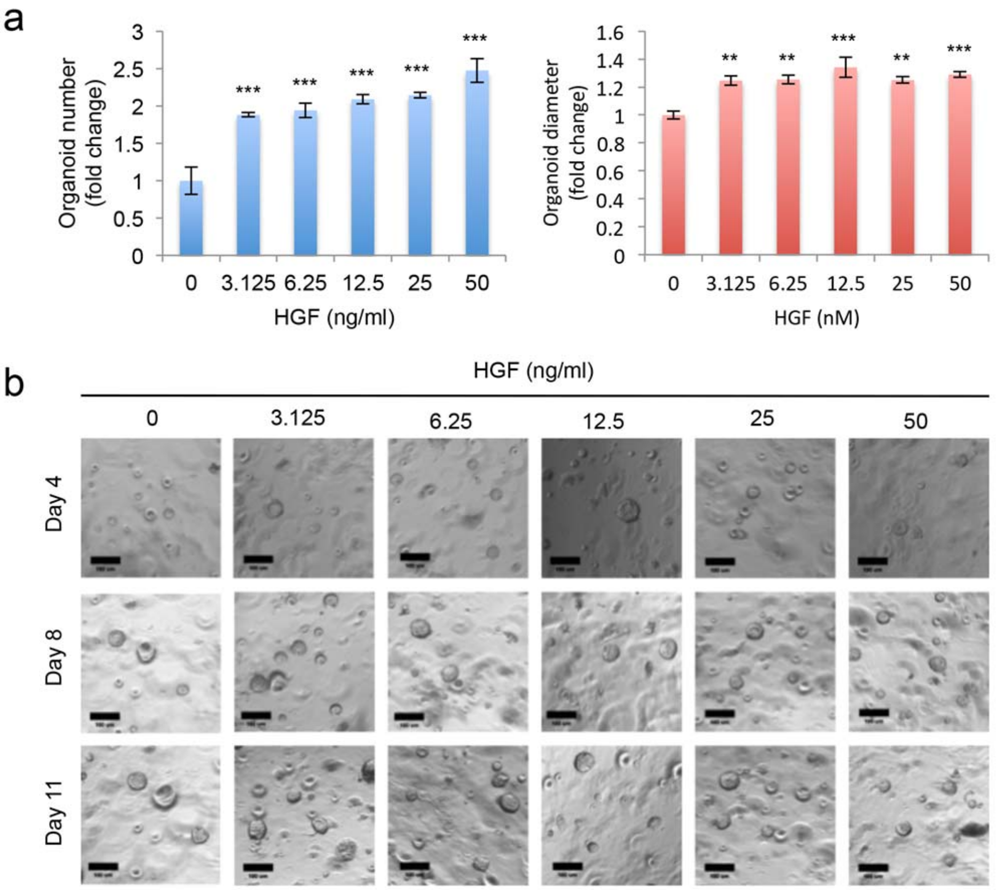

Supplementary Figure 24: HGF supplementation increases organoid forming efficiency and organoid diameter. Mammary epithelial cells were freshly isolated, embedded in matrigel and exposed to culture medium containing Neuregulin 1 (100ng/ml), Noggin (100ng/ml), R-spondin 1 (2.656 ng/ml) and increasing concentrations of HGF. (a) After 11 days, the number and diameter of organoids was assessed using GelCount^{TM} scanner and software from Oxford Optronix. Data are expressed as fold change (vs untreated, n=3, means±s.e.m.). **, $p < 0.01$; ***, $p < 0.001$, ANOVA with Dunnett's post-hoc comparison. (b) Representative pictures of mammary organoids cultured for 4, 8 and 11 days in the presence of increasing concentrations of HGF. Scale bar, 100 μm.

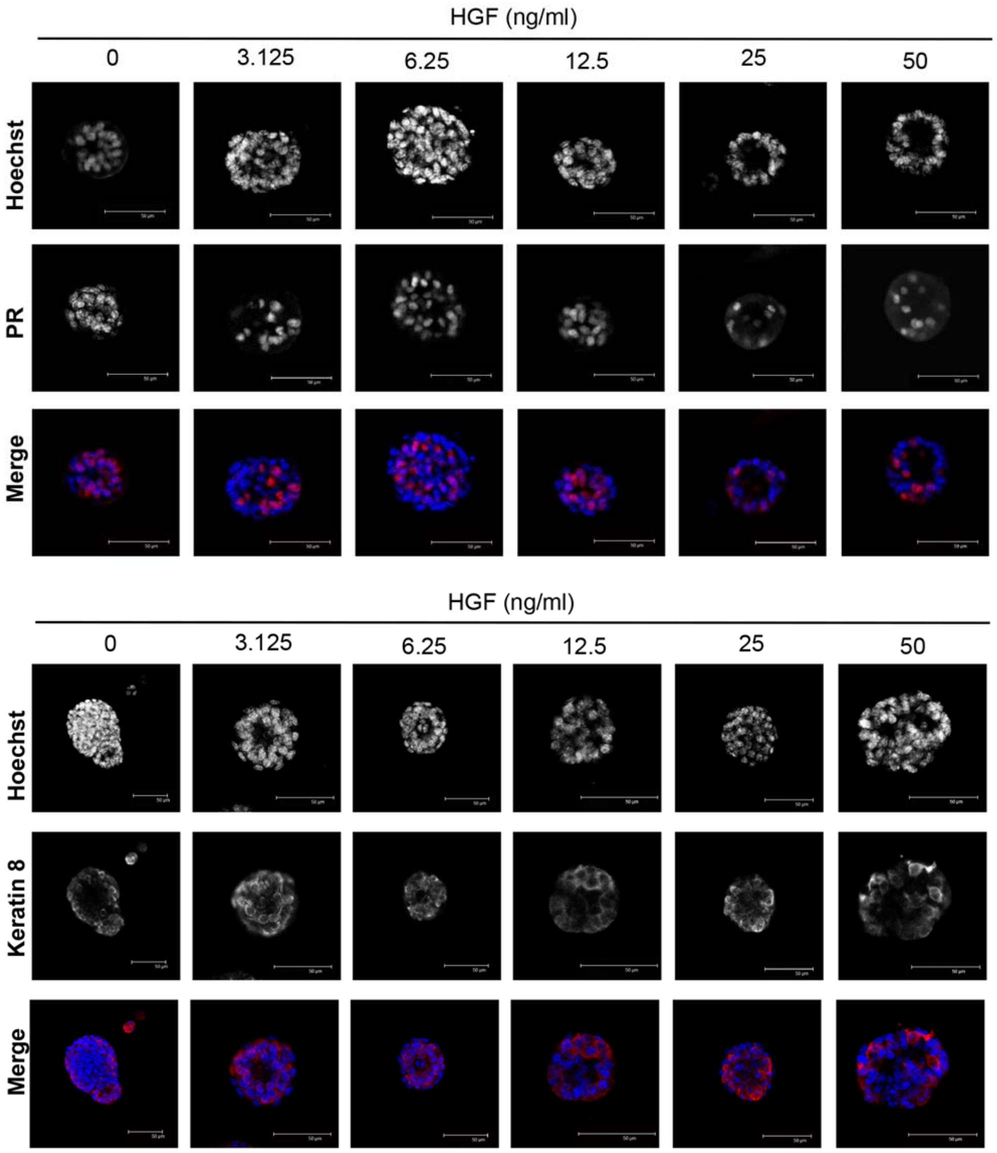

Supplementary Figure 25: HGF supplementation does not alter luminal markers localisation. Mammary epithelial cells were freshly isolated, embedded in matrigel and exposed to culture medium containing Neuregulin 1 (100ng/ml), Noggin (100ng/ml), R-spondin 1 (2.656 ng/ml) and increasing concentrations of HGF. After 14 days in culture, mammary organoids were fixed and stained for luminal markers progesterone receptor (PR) and keratin 8 and for the nuclear marker hoechst. Scale bar, 50 µm.

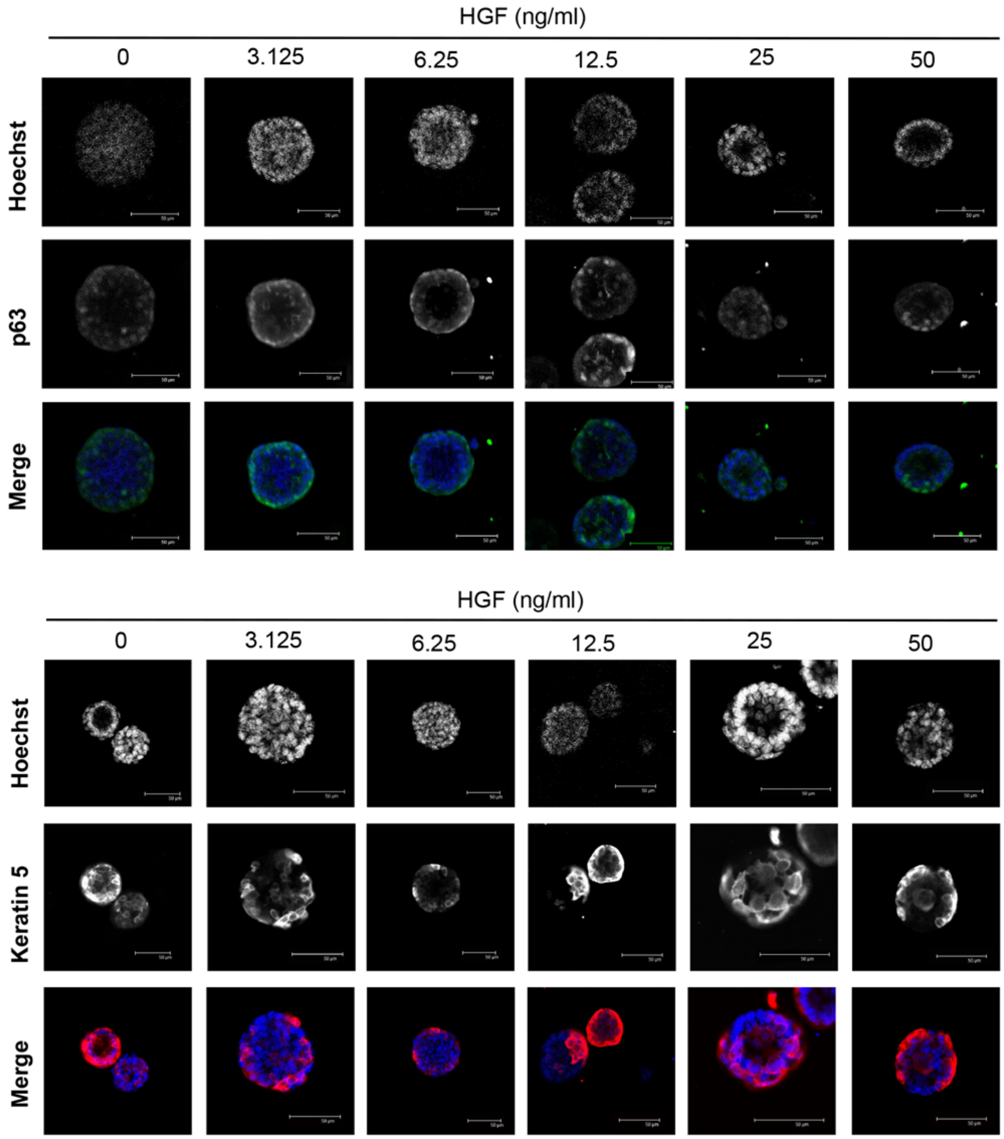

Supplementary Figure 26: HGF supplementation does not alter basal markers localisation. Mammary epithelial cells were freshly isolated, embedded in matrigel and exposed to culture medium containing Neuregulin 1 (100ng/ml), Noggin (100ng/ml), R-spondin 1 (2.656 ng/ml) and increasing concentrations of HGF. After 11 days in culture, mammary organoids were fixed and stained for basal markers p63 and keratin 5 and for the nuclear marker hoechst. Scale bar, 50 µm.

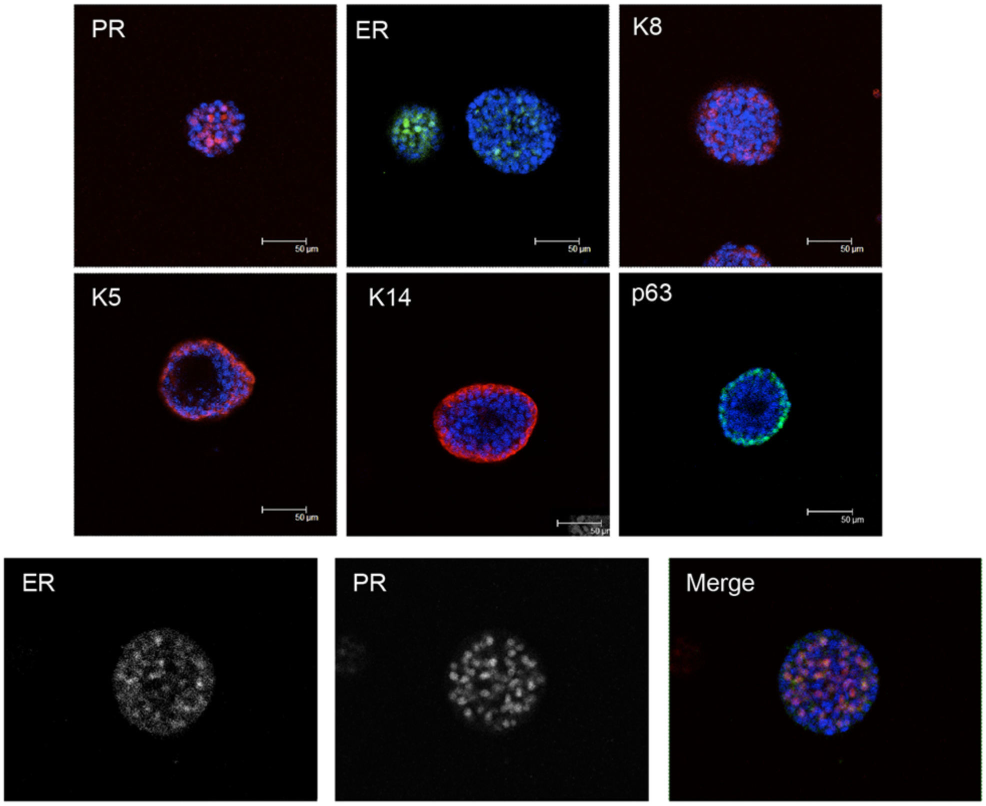

Supplementary Figure 27: Cellular characterisation of mammary organoids originating from single CD24^{high} Sca1^{-} cells. Single CD24^{high} Sca1^{-} cells were cultured for 14 days with R-spondin 1 (2.6 ng/ml), Neuregulin 1 (100 ng/ml) and Noggin (100 ng/ml). Organoids were fixed and stained for luminal markers (progesterone receptor, PR; estrogen receptor, ER; keratin 8, K8) and basal markers (keratin 5, K5; keratin 14, K14; p63). Counterstain, hoechst (blue). Scale bars, 50 µm.

Supplementary Table 1: Composition of B27 supplement

Reagent	Supplier, catalog number	Concentration (μg/ml)
Albumin	Gift from Dr Tess Saltmarsh	2500
Catalase	Sigma-Aldrich, C41	2.5
Glutathione (reduced)	Sigma-Aldrich, G6013	1
Insulin	Sigma-Aldrich, I1882	4
Superoxide dismutase	Sigma-Aldrich, S5395	2.5
Holo-transferrin	Calbiochem, 616242	5
Triiodo-L-thyronine (T3)	Sigma-Aldrich, T6397	0.002
L-carnitine	Sigma-Aldrich, C7518	2
Ethanolamine	Sigma-Aldrich, E9508	1
D-galactose	Sigma-Aldrich, G0625	15
Putrescine	Sigma-Aldrich, P5780	16.1
Sodium selenite	Sigma-Aldrich, S9133	0.01435
Corticosterone	Sigma-Aldrich, C2505	0.02
Linoleic acid	Sigma-Aldrich, L1012	1
Linolenic acid	Sigma-Aldrich, L2376	1
α-tocopherol	Sigma-Aldrich, 95240	1
α-tocopherol acetate	Sigma-Aldrich, T3001	1
Biotin	Sigma-Aldrich, B4639	0.1

Supplementary Table 2: Composition of N2 supplement

Reagent	Supplier, catalog number	Concentration (µg/ml)
Holo-transferrin	Calbiochem, 616242	10 000
Insulin	Sigma-Aldrich, I1882	500
Putrescine	Sigma-Aldrich, P5780	1611
Sodium selenite	Sigma-Aldrich, S9133	0.52

Supplementary Table 3: Karyotype analysis of organoids grown under the 3 culture conditions for 1 month (early), 2.5 months (mid) and 4 months (late). Chromosomal spreads with 40±1 chromosomes were considered normal.

	**Nrg1**			**EGF/high R-spondin 1**			**Nrg1/low R-spondin 1**		
	Analysed cells	Normal	Abnormal	Analysed cells	Normal	Abnormal	Analysed cells	Normal	Abnormal
**Early**	NA	NA	NA	7	5	2 (38)	11	10	1 (38)
					71%	29%		91%	9%
**Mid**	11	10	1	NA	NA	NA	15	13	2
		91%	9%					87%	13%
**Late**	NA	NA	NA	26	3	23	29	4	25
					12%	88%		14%	86%

Supplementary Table 4: Composition of immunofluorescence buffer

Reagent	Supplier, catalog number	Final % in PBS (Invitrogen)
Albumin	Gift from Dr Tess Saltmarsh	0.1
Triton X-100	Sigma-Aldrich, T9284	0.2
Tween 20	Sigma-Aldrich, P1379	0.05

Supplementary Table 5: Antibodies used in this study

Antigen	Source	Name/clone; Catalog number	Incubation and dilution
Keratin 5	Covance	Rabbit polyclonal, PRB-160P	O/N 4°C, 1/100
Keratin 14	Covance	Rabbit polyclonal, PRB-155P	O/N 4°C, 1/500
p63	Abcam	Mouse monoclonal (C-04), ab735	O/N 4°C, 1/40
SMA	Abcam	Rabbit polyclonal, ab5694	O/N 4°C, 1/200
Keratin 8	Abcam	Rabbit polyclonal, ab59400	O/N 4°C, 1/100
PR	Thermo Scientific	Rabbit monoclonal (SP2), RM-9102	O/N 4°C, 1/50
ERα	Dako	Mouse monoclonal (C-1D5), M7047	O/N 4°C, 1/100
β-catenin	Cell Signaling Technology	Rabbit monoclonal (6B3), 9582	O/N 4°C, 1/100
E-cadherin	Abcam	Mouse monoclonal, ab610182	O/N 4°C, 1/400
Ki-67	Millipore	Rabbit polyclonal, AB9260	O/N 4°C, 1/500
BrdU	Sigma-Aldrich	Mouse monoclonal (BU33), B8434	O/N 4°C, 1/500
NKCC1	Dr Jim Turner, NIH, Bethesda, MD	Rabbit polyclonal	O/N 4°C, 1/1000

Supplementary Table 6: Primers used in this study

	Sequence	
Gene	Forward	Reverse
ERα	ATGAAAGGCGGCATACGGAAAG	CACCCATTTCATTTCGGCCTTC
PR	GCTTGCATGATCTTGTGAAACAGC	GGAAATTCCACAGCCAGTGTCC
RankL	GCAGATTTGCAGGACTCGACT	CCCCACAATGTGTTGCAGTT
Wnt4	GTCAGGATGCTCGGACAACAT	CACGTCTTTACCTCGCAGGA
B2M	CTTTCTGGTGCTTGTCTCACTG	AGCATTTGGATTTCAATGTGAG
HPRT	CCTAAGATGAGCGCAAGTTGAA	CCACAGGACTAGAACACCTGCTAA
RPL13A	CACTCTGGAGGAGAAACGGAAGG	GCAGGCATGAGGCAAACAGTC
